# Genome-wide analysis of the rice and arabidopsis *non-specific lipid transfer protein *(*nsLtp*) gene families and identification of wheat *nsLtp *genes by EST data mining

**DOI:** 10.1186/1471-2164-9-86

**Published:** 2008-02-21

**Authors:** Freddy Boutrot, Nathalie Chantret, Marie-Françoise Gautier

**Affiliations:** 1 UMR1098 Développement et Amélioration des Plantes, INRA, F-34060 Montpellier, France; 2 UMR1097 Diversité et Adaptation des Plantes Cultivées, INRA, F-34130 Mauguio, France; 3 The Sainsbury Laboratory, John Innes Centre, Colney Lane, Norwich, NR4 7UH, UK

## Abstract

**Background:**

Plant non-specific lipid transfer proteins (nsLTPs) are encoded by multigene families and possess physiological functions that remain unclear. Our objective was to characterize the complete *nsLtp *gene family in rice and arabidopsis and to perform wheat EST database mining for *nsLtp *gene discovery.

**Results:**

In this study, we carried out a genome-wide analysis of *nsLtp *gene families in *Oryza sativa *and *Arabidopsis thaliana *and identified 52 rice *nsLtp *genes and 49 arabidopsis *nsLtp *genes. Here we present a complete overview of the genes and deduced protein features. Tandem duplication repeats, which represent 26 out of the 52 rice *nsLtp *genes and 18 out of the 49 arabidopsis *nsLtp *genes identified, support the complexity of the *nsLtp *gene families in these species. Phylogenetic analysis revealed that rice and arabidopsis nsLTPs are clustered in nine different clades. In addition, we performed comparative analysis of rice *nsLtp *genes and wheat (*Triticum aestivum*) EST sequences indexed in the UniGene database. We identified 156 putative wheat *nsLtp *genes, among which 91 were found in the 'Chinese Spring' cultivar. The 122 wheat non-redundant nsLTPs were organized in eight types and 33 subfamilies. Based on the observation that seven of these clades were present in arabidopsis, rice and wheat, we conclude that the major functional diversification within the nsLTP family predated the monocot/dicot divergence. In contrast, there is no type VII nsLTPs in arabidopsis and type IX nsLTPs were only identified in arabidopsis. The reason for the larger number of *nsLtp *genes in wheat may simply be due to the hexaploid state of wheat but may also reflect extensive duplication of gene clusters as observed on rice chromosomes 11 and 12 and arabidopsis chromosome 5.

**Conclusion:**

Our current study provides fundamental information on the organization of the rice, arabidopsis and wheat *nsLtp *gene families. The multiplicity of nsLTP types provide new insights on arabidopsis, rice and wheat *nsLtp *gene families and will strongly support further transcript profiling or functional analyses of *nsLtp *genes. Until such time as specific physiological functions are defined, it seems relevant to categorize plant nsLTPs on the basis of sequence similarity and/or phylogenetic clustering.

## Background

Plant non-specific lipid transfer proteins (nsLTPs) were first isolated from spinach leaves and named for their ability to mediate the *in vitro *transfer of phospholipids between membranes [1]. NsLTPs are widely distributed in the plant kingdom and form multigenic families of related proteins. However, *in vitro *lipid transfer or binding has been demonstrated only for a limited number of proteins and most nsLTPs have been identified on the basis of sequence homology, sequences deduced from cDNA clones or genes. All known plant nsLTPs are synthesized as precursors with a N-terminal signal peptide. Plant nsLTPs are small (usually 6.5 to 10.5 kDa) and basic (isoelectric point (pI) ranging usually from 8.5 to 12) proteins characterized by an eight cysteine motif (8 CM) backbone as follows: C-Xn-C-Xn-CC-Xn-CXC-Xn-C-Xn-C [2]. The cysteine residues are engaged in four disulfide bonds that stabilize a hydrophobic cavity, which allows the binding of different lipids and hydrophobic compounds *in vitro *[3]. Based on their molecular masses, plant nsLTPs were first separated into two types: type I (9 kDa) and type II (7 kDa) that are distinct both in terms of primary sequence identity (less than 30%) and lipid transfer efficiency [3]. Although they have different cysteine pairing patterns, type I and type II nsLTPs constitute a structurally related family of proteins. Type I nsLTPs are characterized by a long tunnel-like cavity [4,5] while a wheat type II nsLTP has two adjacent hydrophobic cavities [6]. Several anther-specific proteins that display considerable homology with plant nsLTPs [7] have been proposed as a third type that differs from the two others by the number of amino acid residues interleaved in the 8 CM structure [8]. To date, no structural data exists on the lipid transfer ability of type III nsLTPs.

Because they have been shown to transfer lipid molecules between membranes *in vitro*, plant nsLTPs were first suggested to be involved in membrane biogenesis [1]. However, as they are synthesized with a N-terminal signal peptide [9], nsLTPs could not fulfill this function and were thought to be involved in secretion of extracellular lipophillic material, including cutin monomers [10]. NsLTPs are possibly involved in a range of other biological processes, but their physiological function is not clearly understood. Like many other families of low molecular mass cysteine-rich proteins, nsLTPs display intrinsic antimicrobial properties and are thought to participate in plant defense mechanisms [11,12]. This hypothetical function is also supported by the induction of the expression of many *nsLtp *genes in response to biotic infections or application of fungal elicitors [13-17] and by the enhanced tolerance to bacterial pathogens by overexpression of a barley *nsLtp *gene in transgenic arabidopsis [18]. Due to their possible involvement in plant defense mechanisms, nsLTPs are recognized to be pathogenesis-related proteins and constitute the PR-14 family [19]. Roles in plant defense signaling pathways have also been proposed since the disruption of the arabidopsis *DIR1 *gene, which encodes a nsLTP with an 8 CM distinct from those of types I, II or III, impairs the systemic acquired resistance signaling pathway [20]. Similarly a wheat nsLTP competes with the fungal cryptogein for a same binding site in tobacco plasma membranes [21]. A role in the mobilization of lipid reserves has also been suggested for germination-specific nsLTPs [22-24]. Finally, nsLTPs are thought to possess a function in male reproductive tissues [25]. This role appears to be mainly related to type III nsLTPs whose genes display anther-specific expression [7], and to a few type I *nsLtp *genes including the rape *E2 *gene [25], the arabidopsis *AtLtp12 *gene (*At3g51590*) [26] and the rice *t42 *gene (*Os01g12020*) [27] that are also predominantly expressed at the early stage of anther development. It has been suggested that nsLTPs are involved in the deposition of material in the developing pollen wall [25]; however their precise function in pollen remains to be elucidated.

Plant nsLTPs are encoded by small multigene families but to date none has been extensively characterized. Six members have been identified in pepper [28], 11 in cotton [29], 14 in loblolly pine [30], 15 in arabidopsis [31], and 23 in wheat [32]. The availability of the complete sequence of the arabidopsis [33], rice for both *indica *[34] and *japonica *subspecies [35], poplar [36] and grapevine [37] genomes has greatly enhanced our ability to characterize complex multigene families [38-40]. In polyploid genomes such as the allohexaploid wheat *Triticum aestivum*, the presence of multiple putative copies of each gene increases the complexity of the multigene families and the number of closely related sequences. With around 16,000 Mb [41], the genome of the hexaploid wheat is 128 times the size of the genome of the dicotyledonous model plant *Arabidopsis thaliana *and 38 times that of the monocotyledonous model plant *Oryza sativa *and has not been sequenced yet. Nevertheless, efforts made to generate wheat cDNA libraries [42-45] mean EST database mining can also be a successful strategy for the identification of multigene family members in complex genomes [46,47]. In wheat, novel genes encoding polyphenol oxidases [48], storage proteins [49] and nsLTPs [50] were identified by EST database mining.

In the present study, we took advantage of the completion of the rice (*japonica *subspecies) and arabidopsis genome sequences to perform a genome-wide analysis of the *nsLtp *gene family in both species. In an effort to identify new members of the wheat *nsLtp *gene family, we searched the large public-domain collection of wheat ESTs for sequences displaying homologies with characterized rice *nsLtp *genes. In order to compare rice, arabidopsis and wheat nsLTP evolution, we performed phylogenetic analysis of the nsLTPs from these three plant species.

## Results

### The *Oryza sativa nsLtp *gene family is composed of 52 members

Based on a conserved 8 CM, nsLTPs remain a structurally-related family of proteins. However, as a structural scaffold, this motif is also found in several plant protein families that are clustered in a single family (protease inhibitor/seed storage/LTP family) in the Pfam collection of protein families and domains [51]. In order to identify the complete and non-redundant set of *nsLtp *genes in rice, we conducted an *in silico *analysis of the *Oryza sativa *subsp. *japonica *'Nipponbare' genome. At the time of this study (November 2006), the Gramene database contained 101 genomic sequences annotated putative rice *nsLtp *genes. Each of the deduced protein sequences was manually assessed through the analysis of the cysteine residue patterns. The diversity of the retrieved 8 CM proteins enabled several cell wall glycoproteins to be distinguished including 23 glycosylphosphatidylinositol-anchored proteins characterized by a specific C-terminal sorting sequence [52], 21 proline-rich proteins and hybrid proline-rich proteins characterized by a high proportion of proline, histidine and glycine residues in the sequence comprised between the signal peptide and the 8 CM [53], and one glycine-rich protein [54] (Additional file 1). All these sequences displayed a supplementary motif (described above) not present in nsLTPs and were thus discarded. Other proteins were also discarded; they consist of three alpha-amylase/trypsin inhibitors which contain 10 cysteine residues engaged in five disulfide bonds [55], three prolamin storage proteins which lack the CXC motif and two 2S albumin storage proteins which present a molecular mass (MM) of about 20 kDa. Additionally, we eliminated two probable pseudogenes that have no corresponding transcripts indexed in the GenBank database and display mutation accumulations that result in the absence of the CC motif (Os04g09520) or a truncated 5' exon that curtails the signal peptide sequence (Os02g24720). As a result, only 46 out of the 101 genomic sequences initially annotated as putative *nsLtp *genes were found to encode proteins displaying the features of plant nsLTPs (Table 1). In addition to the presence of a signal peptide and the 8 CM (C-Xn-C-Xn-CC-Xn-CXC-Xn-C-Xn-C), the major feature we observed was a generally small MM (6.5 to 10.5 kDa), criteria that were those of type I and II nsLTPs described as having a lipid transfer activity [1,56].

**Table 1:** *NsLtp *genes identified in the *Oryza sativa *subsp. *japonica *genome and features of the deduced proteins. Identical proteins refer to their relative redundant form. A cluster of tandem duplication repeats is indicated by a vertical line before the gene names (see also Figure 1).

*nsLtp *gene	locus/model	intron	signal peptide	mature protein		
		
		bp	AA	AA	MM	pI ^a^
**Type I**						
*OsLtpI.1*	Os01g12020.1	103	24	99	10212	4.36
*OsLtpI.2*	Os01g60740 ^b^	86	27	93	9464	10.55
*OsLtpI.3*	Os03g59380.1	94	33	91	9085	12.07
*OsLtpI.4*	Os05g40010.1	372	30	99	9780	12.05
*OsLtpI.5*	Os06g06340.1	100	28	98	10069	9.84
*OsLtpI.6*	Os06g34840.1	2740	27	120	12297	3.92
*OsLtpI.7*	Os08g03690.1	547	27	93	9621	9.90
|*OsLtpI.8*	Os11g02330 ^c^	106	27	92	9336	10.25
|*OsLtpI.9*	Os11g02350.1	90	28	93	9437	10.89
|*OsLtpI.10*	Os11g02379.1 ^d^	114	25	91	8895	11.81
|*OsLtpI.11*	Os11g02379.2	89	26	92	8916	10.55
|*OsLtpI.12*	Os11g02400.1	106	26	92	9031	11.50
|*OsLtpI.13*	Os11g02424.2	709	26	92	9104	11.50
*OsLtpI.14*	Os11g24070.1	116	25	92	9147	12.20
|*OsLtpI.15*	Os12g02290.1	133	27	OsLTPI.8		
|*OsLtpI.16*	Os12g02300.1	90	OsLTPI.9			
|*OsLtpI.17*	Os12g02310.1	102	25	92	8930	10.55
|*OsLtpI.18*	Os12g02320.1	138	25	91	8909	11.81
|*OsLtpI.19*	Os12g02330.1	106	26	OsLTPI.12		
|*OsLtpI.20*	Os12g02340.1	713	26	OsLTPI.13		
**Type II**						
|*OsLtpII.1*	Os01g49640.1	none	26	77	8119	11.98
|*OsLtpII.2*	Os01g49650.1	none	36	76	7987	11.28
*OsLtpII.3*	Os03g02050.1	none	20	76	7549	11.90
*OsLtpII.4*	Os05g47700.1	none	27	67	7066	10.16
*OsLtpII.5*	Os05g47730.1	none	27	69	7270	10.66
*OsLtpII.6*	Os06g49190.1	none	27	67	6967	10.64
|*OsLtpII.7*	Os10g36070.1	none	26	74	7613	9.84
|*OsLtpII.8*	Os10g36090.1	none	26	74	7659	9.84
|*OsLtpII.9*	Os10g36100 ^e^	none	26	75	7774	9.84
|*OsLtpII.10*	Os10g36110.1	none	25	75	7926	9.84
|*OsLtpII.11*	Os10g36160.1	none	25	69	7382	7.06
|*OsLtpII.12*	Os10g36170.1	none	24	67	6890	11.90
*OsLtpII.13*	Os11g40530.1	none	36	74	7665	12.14
**Type III**						
*OsLtpIII.1*	Os08g43290.1	84	26	68	6744	7.84
*OsLtpIII.2*	Os09g35700.1	107	26	69	6839	7.84
**Type IV**						
|*OsLtpIV.1*	Os01g68580.1	none	29	82	8908	10.65
|*OsLtpIV.2*	Os01g68589.1	none	25	78	8291	9.90
*OsLtpIV.3*	Os07g18750.1	none	28	76	8073	7.84
*OsLtpIV.4*	Os07g18990.1	none	23	81	8420	9.86
**Type V**						
*OsLtpV.1*	Os01g62980.1	97	27	91	9390	12.05
|*OsLtpV.2*	Os04g33920.1	290	22	94	9608	10.22
|*OsLtpV.3*	Os04g33930.2	419	26	97	9940	11.28
*OsLtpV.4*	Os05g06780.1	676	24	93	9497	9.69
**Type VI**						
|*OsLtpVI.1*	Os01g58650.1	2851	20	103	10909	4.48
|*OsLtpVI.2*	Os01g58660.1	92	23	89	9876	9.45
*OsLtpVI.3*	Os10g05720.2	440	28	81	8724	9.56
*OsLtpVI.4*	Os11g29420.1	791	29	96	10176	6.00
**Type VII**						
*OsLtpVII.1*	Os11g37280.1	595	27	105	10781	5.32
**Type VIII**						
*OsLtpVIII.1*	Os06g49770 ^f^	221	30	102	9594	9.79
**nsLTPY**						
*OsLtpY.1*	Os03g44000.1	1088	24	109	12073	9.69
*OsLtpY.2*	Os07g27940.1	148	27	107	10892	11.98
*OsLtpY.3*	Os11g34660 ^g^	825	27	104	11394	5.50

AA, number of amino acids; MM, molecular mass in Dalton; pI, isoelectric point.

^a ^cysteine residues were not taken into account in the pI calculation.

^b ^using the transcript structure Os01g60740.2.

^c ^annotations curated (strand: +1; exon 1 start: 679124, end: 679473; exon 2 start: 679580, end: 679589).

^d ^annotations curated (strand: +1; exon 1 start: 702105, end: 702445; exon 2 start: 702560, end: 702569).

^e ^annotations curated (strand: +1; exon start: 18974249, end: 18974554).

^f ^annotations curated (strand: +1; exon 1 start: 30113033, end: 30113426; exon 2 start: 30113648, end: 30113652).

^g ^annotations curated (strand: +1; exon 1 start: 19789864, end: 19790209; exon 2 start: 19791035, end: 19791084).

Next, a search for misannotated putative *nsLtp *genes was performed by blastn and tblastn searches of the TIGR Rice Pseudomolecules [57] using as query sequences the 46 rice genes and the 35 previously identified wheat nsLTPs and *nsLtp *genes [32]. This approach resulted in the identification of six additional putative *nsLtp *genes leading to a total of 52 rice *nsLtp *genes (Table 1). These new genes were originally not annotated as putative *nsLtp *genes (Os01g58660, Os03g44000, Os09g35700, Os11g02424) or the presence of a frame shift in the coding region failed to identify the deduced proteins as putative nsLTPs (Os11g02330, Os11g02379.1).

### The *Arabidopsis thaliana nsLtp *gene family is composed of 49 members

The same approach was used for arabidopsis. Locus annotations and protein domain descriptions allowed the identification of 112 loci that potentially encode nsLTPs. Analysis of protein primary sequences indicated that 31 of them encode glycosylphosphatidylinositol-anchored proteins, 25 encode hybrid proline-rich proteins and five encode 2S albumin storage proteins that were eliminated (Additional file 1). Three other loci were also discarded since the corresponding deduced protein failed to present an 8 CM (At1g21360, At2g33470, At3g21260). As a result, only 48 out of the 112 loci were found to encode putative nsLTPs (Table 2). Finally, blastn and tblastn searches allowed us to identify one new locus (At1g52415) that encodes an 8 CM protein with no homology with known Pfam domains.

**Table 2:** *NsLtp *genes identified in the *Arabidopsis thaliana *genome and features of the deduced proteins. A cluster of tandem duplication repeats is indicated by a vertical line before the gene names (see also Figure 1).

*nsLtp *gene	locus/model	intron	signal peptide	mature protein		
		bp	AA	AA	MM	pI ^a^

**TypeI**						
*AtLtpI.1*	At2g15050.2	653	25	90	9489	12.13
*AtLtpI.2*	At2g15325.1	127	27	94	10312	4.83
*AtLtpI.3*	At2g18370.1	438	24	92	9092	4.36
|*AtLtpI.4*	At2g38530.1	111	23	95	9661	11.90
|*AtLtpI.5*	At2g38540.1	none	25	93	9281	11.50
*AtLtpI.6*	At3g08770.1	94	19	94	9883	9.61
|*AtLtpI.7*	At3g51590.1	467	24	95	9945	9.61
|*AtLtpI.8*	At3g51600.1	107	25	93	9891	12.68
*AtLtpI.9*	At4g33355.1	112	28	91	9514	9.86
*AtLtpI.10*	At5g01870.1	94	22	94	9923	10.45
|*AtLtpI.11*	At5g59310.1	138	23	89	8854	10.76
|*AtLtpI.12*	At5g59320.1	94	23	92	9221	10.76
**TypeII**						
|*AtLtpII.1*	At1g43665 ^b^	none	22	75	8367	9.59
|*AtLtpII.2*	At1g43666.1	none	19	77	8458	9.67
|*AtLtpII.3*	At1g43667.1	none	21	77	8488	9.59
*AtLtpII.4*	At1g48750.1	none	26	68	7258	10.74
*AtLtpII.5*	At1g66850.1	none	24	78	7970	7.12
*AtLtpII.6*	At1g73780.1	none	29	69	7674	9.67
*AtLtpII.7*	At2g14846.1	none	21	78	8386	9.69
*AtLtpII.8*	At3g12545 ^c^	none	25	64	7206	10.50
*AtLtpII.9*	At3g18280.1	none	28	68	7372	12.40
*AtLtpII.10*	At3g29105 ^d^	none	24	70	7841	9.92
*AtLtpII.11*	At3g57310.1	none	24	79	8504	9.71
|*AtLtpII.12*	At5g38160.1	none	24	79	8309	5.43
|*AtLtpII.13*	At5g38170.1	none	24	79	8342	7.12
|*AtLtpII.14*	At5g38180.1	none	24	71	8127	7.28
|*AtLtpII.15*	At5g38195.1	none	24	71	7718	4.40
**TypeIII**						
*AtLtpIII.1*	At5g07230.1	120	24	67	6883	4.29
*AtLtpIII.2*	At5g52160.1	none	32	64	6791	4.64
*AtLtpIII.3*	At5g62080.1	315	30	65	6636	4.14
**TypeIV**						
|*AtLtpIV.1*	At5g48485.1	none	26	76	7974	4.25
|*AtLtpIV.2*	At5g48490.1	none	25	76	8078	4.59
|*AtLtpIV.3*	At5g55410.1	81	30	77	8544	10.35
|*AtLtpIV.4*	At5g55450.1	none	30	74	7779	9.95
|*AtLtpIV.5*	At5g55460.1	106	32	77	8303	10.50
**TypeV**						
*AtLtpV.1*	At2g37870.1	96	23	92	9575	12.67
*AtLtpV.2*	At3g53980.1	99	23	91	9362	9.91
*AtLtpV.3*	At5g05960.1	88	25	91	9530	10.85
**TypeVI**						
*AtLtpVI.1*	At1g32280.1	258	23	89	9383	9.69
*AtLtpVI.2*	At4g30880.1	192	22	87	9222	9.91
*AtLtpVI.3*	At4g33550 ^e^	79	29	86	9283	10.01
*AtLtpVI.4*	At5g56480.1	150	23	90	9582	4.91
**TypeVIII**						
*AtLtpVIII.1*	At1g70250 ^f^	none	19	90	9865	4.64
**Type IX**						
*AtLtpIX.1*	At3g07450.1	none	29	77	7980	12.16
*AtLtpIX.2*	At3g52130.1	none	26	99	10484	4.40
**nsLTPY**						
*AtLtpY.1*	At1g52415 ^g^	170	24	92	10825	10.25
*AtLtpY.2*	At1g64235 ^h^	577	24	94	10313	10.83
*AtLtpY.3*	At4g08530 ^i^	none	22	104	11859	9.53
*AtLtpY.4*	At4g28395 ^j^	74, 121 ^k^	20	120	13430	5.28

AA, number of amino acids; MM, molecular mass in Dalton; pI, isoelectric point.

^a ^cysteine residues were not taken into account in the pI calculation.

^b ^annotations curated (strand: -1; exon start: 16455949, end: 16456244).

^c ^annotations curated (strand: -1; exon start: 3977557, end: 3977828).

^d ^annotations curated (strand: +1; exon start: 11082271, end: 11082557).

^e ^annotations curated (strand: +1; exon 1 start: 16134443, end: 16134767; exon 2 start: 16134847, end: 16134869).

^f ^annotations curated (strand: +1; exon start: 26456628, end: 26456958).

^g ^annotations curated (strand: +1; exon 1 start: 19529835, end: 19530183; exon 2 start: 19530354, end: 19530355).

^h ^annotations curated (strand: +1; exon 1 start: 23839912, end: 23840250; exon 2 start: 23840828, end: 23840845).

^i ^annotations curated (strand: +1; exon start: 5421971, end: 5422352).

^j ^annotations curated (strand: +1; exon 1 start: 14044281, end: 14044490; exon 2 start: 14044565, end: 14044734; exon 3 start: 14044856, end: 14044898).

^k ^*AtLtpY.4 *contains two introns.

### Organization and structure of the rice and arabidopsis *nsLtp *genes

**Figure 1:**
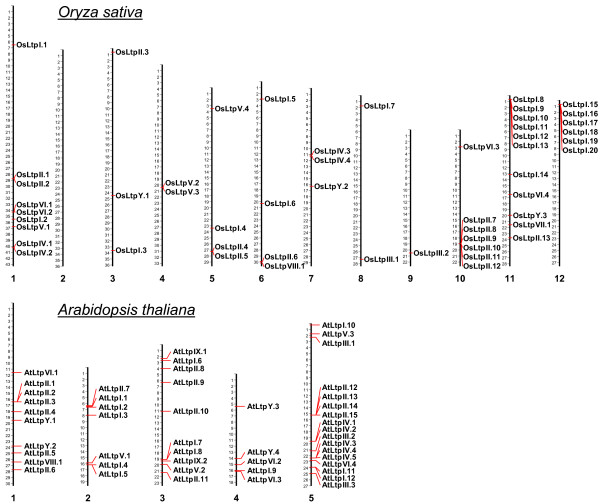
**Organization of *nsLtp *genes in rice and arabidopsis genomes**. Positions of *nsLtp *genes are indicated on chromosomes (scale in Mbp).

Analysis of the physical chromosomal loci revealed that 26 out of the 52 rice *nsLtp *genes and 18 out of the 49 arabidopsis *nsLtp *genes are arranged in tandem duplication repeats (Figure 1). To cover nomenclature in different species, we named rice and arabidopsis *nsLtp *genes encoding nsLTPs *OsLtp *and *AtLtp*, respectively. Genes encoding mature proteins sharing more than 30% identity were grouped in the same type [32]. Genes encoding rice and arabidopsis type I nsLTPs were named *OsLtpI *and *AtLtpI *respectively, and consecutive roman numbers were assigned for the other types.

In rice, two significant clusters of six type I *nsLtp *genes are found on chromosomes 11 and 12. A dot plot alignment of these two clusters clearly showed a co-linear segment that reveals high nucleotide sequence conservation, and indicated homologies between all *nsLtp *genes mainly limited to the ORFs (data not shown). Type II *nsLtp *genes are present as a cluster of six copies repeated in tandem on chromosome 10. Three direct repeat tandems were also identified on chromosome 1 (*OsLtpII.1 *and *OsLtpII.2*; *OsLtpIV.1 *and *OsLtpIV.2*; *OsLtpVI.1 *and *OsLtpVI.2*) and one on chromosome 4 (*OsLtpV.2 *and *OsLtpV.3*). Due to these duplications,*nsLtp *genes are over-represented on rice chromosomes 1, 10, 11 and 12, which carry 33 out of the 52 identified genes. On the contrary, no *nsLtp *genes were identified on chromosome 2.

In arabidopsis, 18 *nsLtp *genes were found organized in seven direct repeat tandems. Whereas one tandem of three repeats is present on chromosome 1 (*AtLtpII.1*, *AtLtpII.2*, and *AtLtpII.3*) and one tandem of two repeats is present on both chromosome 2 (*AtLtpI.4 *and *AtLtpI.5*) and 3 (*AtLtpI.7 *and *AtLtpI.8*), four direct repeat tandems are found on chromosome 5. With two to four repeats, these four tandems lead to the over-representation of *nsLtp *genes on arabidopsis chromosome 5.

With the exception of the *AtLtpIV.3 *and *AtLtpIV.5 *genes, no introns were identified in the coding regions of type II and IV rice and arabidopsis *nsLtp *genes and type IX arabidopsis *nsLtp *genes. On the contrary, all the type I, III, V and VI rice and arabidopsis *nsLtp *genes (except the *AtLtpI.5 *and *AtLtpIII.2 *genes) were predicted to be interrupted by a single intron positioned 2 to 73 bp upstream of the stop codon.

### Identification of *T. aestivum nsLtp *genes by EST database mining

Because the genome of *T. aestivum *has not yet been sequenced, we aimed to identify new members of the wheat *nsLtp *gene family by EST database mining. Since we observed strong homologies between many of the 52 rice *nsLtp *genes, the mismatches consented during the assembly of wheat ESTs in tentative consensus sequences or UniGene clusters (indexed in the TIGR Wheat Gene Index Database and in the NCBI UniGene database, respectively) make these last not appropriate for the identification of novel wheat *nsLtp *genes. Consequently, blast searches were performed against the wheat ESTs indexed in the GenBank database and collected from 239 *T. aestivum *cDNA libraries. To this end, we used the coding sequence of each of the 52 rice *nsLtp *genes listed in Table 1 and each of the 32 wheat genomic and cDNA sequences identified by Boutrot et al. 2007 [32].

ClustalW multiple-sequence alignments were performed for each blastn search. For each new putative wheat *nsLtp *gene identified, additional reiterative blastn searches were performed against the wheat EST database to identify additional related sequences. In total, this survey led to the identification of 156 putative wheat *nsLtp *genes (Table 3 and Additional file 2).

**Table 3:** *Triticum aestivum nsLtp *genes and features of the deduced mature proteins. Details are given in Additional file 2.

*nsLtp *genes			mature nsLTPs		
type	number of subfamilies	number of members	AA	MM	pI ^a^

I	12	85	86–98	8625–9855	4.14, 8.15–11.81
II	8	34	66–71	6841–7437	8.00–11.74
III	2	3	66–71	6727–7107	9.84–10.85
IV	4	12	74–82	7668–8607	11.09
V	3	10	91–99	9240–10514	4.06, 9.54–12.13
VI	2	8	83–94	8608–9793	4.01–4.29, 9.59–9.77
VII	1	3	148–150	15139–15450	9.71–10.39
VIII	1	1	96	9482	4.59

AA, number of amino acids; MM, molecular mass in Dalton; pI, isoelectric point.

^a ^cysteine residues were not taken into account in the pI calculation

We applied to wheat *nsLtp *genes and proteins the nomenclature used for rice and arabidopsis (see above) and the eight types were named *TaLtpI *to *TaLtpVIII*. However, to consider the hexaploid status of the wheat genome we grouped wheat genes into subfamilies of putative homoeologous genes. This was based on the identity matrix (data not shown) calculated from the multiple sequence alignments and the nomenclature criteria that group mature proteins sharing more than 30% identity in a type and more than 75% identity in a subfamily [32]. The 12 type I subfamilies were named *TaLtpIa *to *TaLtpIl*. Finally, the different members of each subfamily were differentiated by consecutive numbers, i.e. *TaLtpIb.1 *to *TaLtpIb.39 *for the 39 members of the type Ib subfamily. The correspondence between the previous nomenclature of wheat *nsLtp *genes [32] and the one used in this paper is shown in Additional file 2.

Since different *T. aestivum *cultivars were used to construct the cDNA libraries, the existence of probable variants of one gene may have resulted in overestimation of *nsLtp *gene diversity. Nevertheless, ESTs corresponding to at least 91 out of the 156 *nsLtp *genes were identified in the *T. aestivum *'Chinese Spring' ('CS') cultivar. The identification of complete subfamily sets in single cultivars, such as the eight members of the *TaLtpVa *subfamily in the 'CS' cultivar, suggests that all the closely related genes of a subfamily reflect recent evolution of paralogous genes. We failed to identify any members of the *TaLtpIe*, *TaLtpIf*, *TaLtpIi*, *TaLtpIk*, *TaLtpIl*, *TaLtpIVd*, *TaLtpVb*, *TaLtpVc*, *TaLtpVIIa *and *TaLtpVIIIa *subfamilies in the 'CS' cultivar. However, most members of these subfamilies were identified in cDNA libraries prepared from specific plant material that were not used to construct 'CS' cDNA libraries.

### Rice, arabidopsis and wheat nsLTP characteristics

The characteristics of the 52 rice and 49 arabidopsis putative nsLTPs are presented in Table 1 and Table 2, respectively. The MM and the theoretical pI of the 122 non-redundant wheat mature nsLTPs are summarized in Table 3 (details in Additional file 2).

Wheat, rice and arabidopsis nsLTPs are synthesized as pre-proteins that contain a putative signal peptide of 16 to 38 amino acids. The putative subcellular targeting of the 257 rice, arabidopsis and wheat nsLTP pre-protein sequences was analyzed using the TargetP 1.1 program and 255 of them present an N-terminal signal sequence that is thought to lead the mature protein through the secretory pathway. TaLTPIVb.3 and TaLTPIl.2 sequences have been predicted to contain a mitochondrial targeting peptide and a signal peptide. But, no conclusion could be drawn about the subcellular localization of these two mature proteins since the reliability of prediction was very weak.

At the pre-protein level, the OsLTPI.9 and OsLTPI.16 deduced proteins are identical. After cleavage of their signal peptide (predicted by the SignalP program), the OsLTPI.8 and OsLTPI.15 mature proteins are identical, as are the OsLTPI.12 and OsLTPI.19 mature proteins and the OsLTPI.13 and OsLTPI.20 mature proteins (Table 1). Therefore, before potential post-translational modifications, the 52 rice *nsLtp *genes encode 48 different mature nsLTPs. The 49 arabidopsis *nsLtp *genes encode proteins that are distinct in both their pre-protein and mature forms (Table 2). Thirty-four wheat proteins are redundant after cleavage of their signal peptide, 15 of them being redundant at the pre-protein level. Therefore, before potential post-translational modifications the 156 wheat putative *nsLtp *genes encode 122 different mature TaLTPs (Additional file 2). The TaLTPIf subfamily displays the strongest conservation since the four members have identical mature protein sequences. A high level of redundancy was also observed in genes of the *TaLtpIg *subfamily since five out of the eight members encode the same TaLTPIg.2 mature protein.

**Figure 2:**
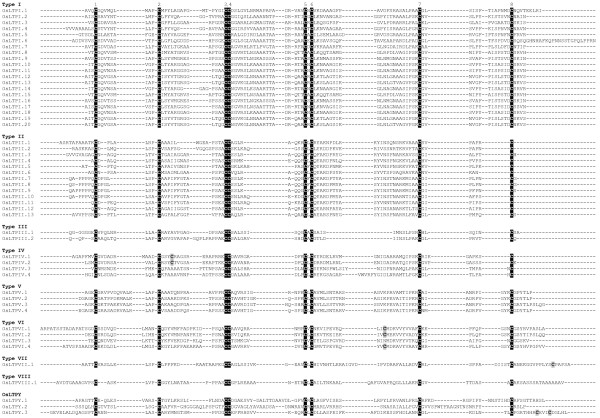
**Multiple sequence alignment of rice nsLTPs**. Amino acid sequences were deduced from *nsLtp *genes identified from the TIGR Rice Pseudomolecules release 4 (Table 1). Sequences were aligned using HMMERalign to maximize the eight-cysteine motif alignment, and manually refined. The conserved cysteine residues are black boxed and additional cysteine residues grey boxed.

**Figure 3:**
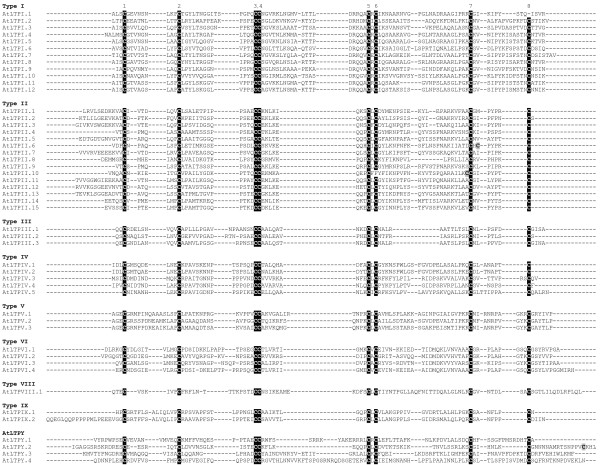
**Multiple sequence alignment of arabidopsis nsLTPs**. Amino acid sequences were deduced from *nsLtp *genes identified from the TAIR arabidopsis genome database (TAIR release 6.0) (Table 2). Sequences were aligned using HMMERalign to maximize the eight-cysteine motif alignment, and manually refined. The conserved cysteine residues are black boxed and additional cysteine residues grey boxed.

**Figure 4:**
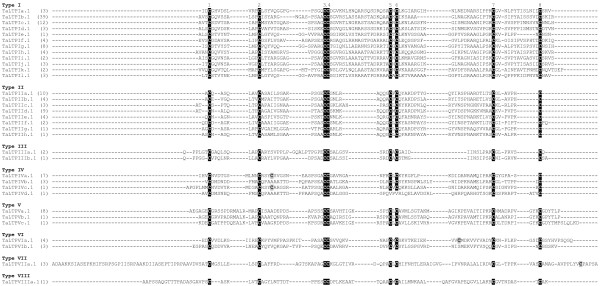
**Multiple sequence alignment of wheat nsLTPs**. Amino acid sequences were deduced from genes or ESTs indexed in the NCBI database. Amino acid sequences were aligned using HMMERalign to maximize the eight-cysteine motif alignment, and manually refined. For each nsLTP subfamily, one sequence is presented and the number of putative members identified is indicated between parentheses. The conserved cysteine residues are black boxed and additional cysteine residues grey boxed. Accession numbers are given in Additional file 2 and amino acid sequence of mature nsLTPs in Additional file 3.

Since it allows all the cysteine residues to be maintained in a conserved position, the HMMalign program was preferred to ClustalW and was thus used to perform the multiple alignments of rice (Figure 2), arabidopsis (Figure 3) and wheat (Figure 4) nsLTPs. Based on the identity matrix (data not shown) calculated from the multiple sequence alignments and the nomenclature criteria that group mature proteins sharing more than 30% identity in a type [32], 49 out of the 52 rice nsLTPs, 45 out of the 49 arabidopsis nsLTPs and the 122 wheat nsLTPs were found to be clustered in nine types. The majority (147 out of 223) of the rice, arabidopsis and wheat *nsLtp *genes encode proteins that belong to the type I and type II nsLTPs. Fourteen rice, 15 arabidopsis and 34 wheat proteins described six new nsLTP types named types IV to IX. Three rice proteins and four arabidopsis proteins display less than 30% identity between themselves or with other nsLTPs to either make a type by themselves or be integrated in an already identified type. Therefore, these proteins were named OsLTPY.1 to OsLTPY.3 and AtLTPY.1 to AtLTPY.4.

Rice, wheat and arabidopsis nsLTPs are small proteins since their MMs usually range from 6636 Da to 10909 Da. However the OsLTPI.6 protein and the three members of the type VII wheat nsLTPs display unusual high MMs (13–15 kDa) due to the presence of supernumerary amino acid residues located at the C-terminal or N-terminal extremity of the deduced mature proteins. While the MM of nsLTPs previously allowed discrimination of the 9 kDa type I and the 7 kDa type II, type III nsLTPs were also found to present a MM of about 7 kDa. With nine nsLTP types identified, the relationship between MM and nsLTP type becomes more complex and is not anymore a good criterion to classify nsLTPs. The majority (199 out of 223) rice, wheat and arabidopsis non-redundant nsLTPs display a basic pI that is another characteristic of nsLTPs. In no case did nsLTPs with an acidic pI (3.92–5.50) form a specific type.

One characteristic of plant nsLTPs types I and II is the absence of tryptophane residues. Although this is usually the case, we found two type I (AtLTPI.2, AtLTPI.10), three type II (OsLTPII.1, AtLTPII.3, AtLTPII.11), four type IV (OsLTPIV.3, AtLTPIV.1, AtLTPIV.2, TaLTPIVb.1) and three nsLTPY proteins (OsLTPY.2, AtLTPY.1, AtLTPY.3) that contain one or two tryptophane residues.

The main characteristic of plant nsLTPs is the presence of eight cysteine residues in a strongly conserved position Cys1-Xn-Cys2-Xn-Cys3Cys4-Xn-Cys5XCys6-Xn-Cys7-Xn-Cys8. All the rice nsLTPs display this feature whereas two arabidopsis and two wheat nsLTPs present a different pattern. The Cys8 is missing in AtLTPI.1 and the Cys6 in AtLTPII.10. The TaLTPIVd.1 lacks Cys5 and Cys6 in the CXC motif and the TaLTPVIa.5 lacks the Cys7. Conversely, the members of the TaLTPIVa subfamilies, TaLTPIVc.1, OsLTPIV.1 and OsLTPIV.2 harbor an additional cysteine residue between Cys2 and Cys3, the TaLTPVIa subfamily members, OsLTPVI.1, OsLTPVI.2 OsLTPVI.4 and AtLTPII.10 between Cys6 and Cys7, AtLTPII.6 after Cys7, and the TaLTPVIIa subfamily members and OsLTPVII.1 after the Cys8 of the 8 CM.

**Figure 5:**
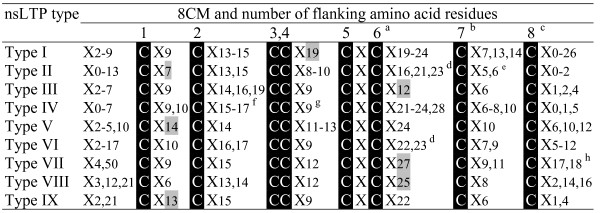
**Diversity of the eight cysteine motif in rice, arabidopsis and wheat nsLTP types**. The consensus motif of each nsLTP type was deduced from the analysis of the matures sequences of the 52 rice nsLTPs, the 49 arabidopsis nsLTPs and the 156 wheat nsLTPs presented in Table 1, Table 2, and Additional file 2, respectively. AtLTPII.8 that appears to be more distantly related to other type II sequences (see the phylogenetic analysis) was excluded. The values allowing direct identification of the nsLTP type are grey boxed. ^a ^cysteine residue number 6 is missing in AtLTPII.10. ^b ^cysteine residue number 7 is missing in TaLTPVIa.5. ^c ^cysteine residue number 8 is missing in AtLTPI.1. ^d ^AtLTPII.10, OsLTPVI.1, OsLTPVI.2, OsLTPVI.4, and TaLTPVIa subfamily members harbor an extra cysteine residue. All type VI contain a Val 4 aa before Cys7 and a Met 10 aa before Cys7 allowing a distinction between type IV and type VI. ^e ^AtLTPII.6 harbors an extra cysteine residue. ^f ^TaLTPIVc.1 and TaLTPIVa subfamily members harbor an extra cysteine residue. ^g ^12 amino acid residues were counted for the TaLTPIVd.1 that displays no CXC motif. ^h ^OsLTPVII.1 and TaLTPVIIa.1 subfamily members harbor an extra cysteine residue.

The multiple alignment of the cysteine motifs of rice, arabidopsis and wheat nsLTPs also revealed a variable number of inter-cysteine amino acid residues (summarized in Figure 5). The AtLTPII.8 which is phylogenetically distant from all other type II *nsLtp *genes (see the phylogenetic analysis below) was not taken into consideration. In this way, seven nsLTP types can be identified through typical spacings for this motif. For example, type I nsLTPs contain 19 residues between the conserved Cys4 and Cys5 residues while types III, VII and VIII contain respectively 12, 27 and 25 residues between the conserved Cys6 and Cys7 residues. Similarly, types II, V and IX can be described with respectively 7, 14 and 13 residues between the conserved Cys1 and Cys2 residues. Only types IV and VI can not be distinguished based on this simple feature. A closer analysis of the sequences indicates that type VI nsLTPs are always characterized by a methionine and a valine residue present 10 and 4 aa before Cys7, respectively (Figures 2, 3, 4). At these positions, these two aa are always different in type IV nsLTPs and allow the direct distinction of type IV and VI nsLTPs.

### Phylogenetic analysis of rice, arabidopsis and wheat nsLTPs

**Figure 6:**
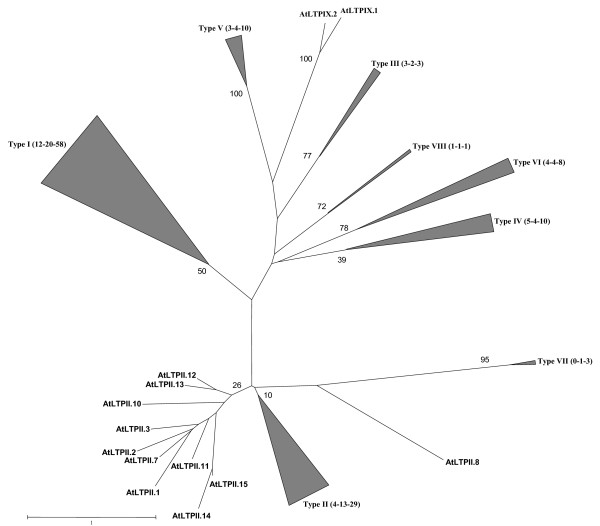
**Unrooted phylogenetic tree between rice, arabidopsis and wheat nsLTP gene families**. The mature sequences of the 122 non-redundant wheat nsLTPs, the 49 rice nsLTPs, and the 45 arabidopsis nsLTPs were aligned using HMMalign and then manually refined. The phylogenetic tree was built from the protein alignment (Additional file 3) with the maximum-likelihood method using the PHYML program [75]. When possible, subtrees including sequences of the same type are grouped and represented by a grey triangle close to which is indicated, in brackets, the number of sequences of arabidopsis, rice and wheat respectively. Subtrees are detailed in Figure 7. Bootstrap values (% of 100 re-sampled data set) are indicated for each node.

**Figure 7:**
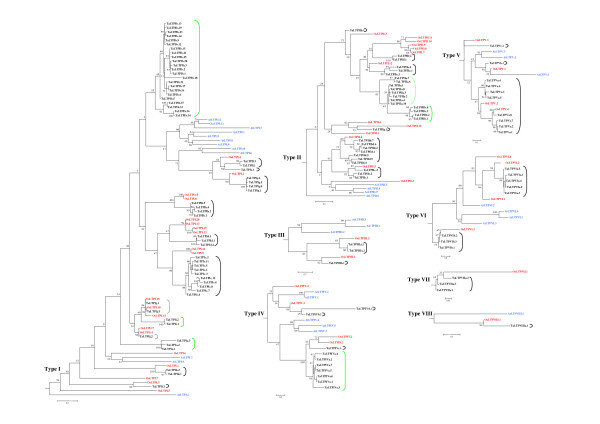
**Rooted phylogenetic subtrees detailed from unrooted phylogenetic tree between rice, arabidopsis and wheat nsLTP gene families**. Each subtree represented by a grey triangle in Figure 6 is detailed and rooted on the remaining parts of the tree. Wheat nsLTPs are in black, rice nsLTPs in red and arabidopsis nsLTPs in blue. Monophyletic subfamilies are indicated by solid brackets, paraphyletic subfamilies by dotted brackets. Black brackets indicate the wheat subfamily in which a potential rice ortholog nsLTP gene is present, and green brackets indicate wheat-specific subfamilies. Bootstrap values (% of 100 re-sampled data set) are indicated for each node.

In order to analyze the phylogenetic organization of the nsLTP families, we constructed a phylogenetic tree from the alignment of respectively 45, 49 and 122 sequences of arabidopsis, rice and wheat nsLTPs, using the maximum-likelihood inference. Redundant mature wheat nsLTPs were eliminated but the arabidopsis and rice complete families were included. The solidity of the nodes was assessed by 100 bootstrap resampling repetitions. The seven arabidopsis and rice nsLTPY proteins were first included but due to the fact that their position was not well supported (nodes with weak bootstrap values) and consequently risked muddling the phylogenetic signal, they were excluded from the alignment. In the first attempt, several cysteine-rich protein sequences (metallothioneins, thionins and defensins from arabidopsis and rice) were tested as potential roots, but their position was different and none were supported by significant bootstrap values. Moreover, the phylogenetic relationships between types were not reliable whatever the root chosen. Consequently, we chose to present the complete condensed unrooted tree (Figure 6) where each of the subtrees (detailed in Figure 7) is rooted by all the other sequences.

The general organisation of the tree is coherent with the classification of nsLTPs in nine types. All the sequences belonging to the same type are grouped and constitute monophyletic groups (i.e. clades) except for type II nsLTPs. The bootstrap values supporting the clades corresponding to types III, V, VI, VII, VIII and IX are high, respectively 77, 100, 78, 95, 72 and 100. Types I and IV have lower bootstrap values, respectively 50 and 39. Based on the criteria that group mature proteins sharing more than 30% identity in a type, AtLTPIX.1 and AtLTPIX.2 were first included in type IV although their identity with other type IV nsLTPs was very low (12.6% to 30.1%). However, according to their position in the phylogenetic tree these sequences probably do not share the same common ancestor as other type IV nsLTPS and were classed in a new type named type IX. Type II nsLTPs are close in the tree but do not constitute a clade. This is mainly due to several *A. thaliana *nsLTPs (AtLTPII.1, AtLTPII.2, AtLTPII.3, AtLTPII.7, AtLTPII.8, AtLTPII.10, AtLTPII.11, AtLTPII.12, AtLTPII.13, AtLTPII.14 and AtLTPII.15), which appear to be more distantly related to other type II sequences. When the tree is built only with wheat and rice sequences, type II nsLTPs appear to be monophyletic and highly supported (bootstrap value 95; data not shown).

The distribution of nsLTPs in the tree is not either quantitatively or qualitatively homogeneous. As can be seen in Figure 7, there are significant differences in the number of sequences, with as few as two sequences for type IX nsLTPs and 90 for type I nsLTPs. Moreover, nsLTPs of each species are not homogeneously distributed within each type. Surprisingly, arabidopsis does not posses any type VII nsLTPs and no type IX nsLTPs were identified in rice and wheat.

Only type VIII nsLTPs displayed the simple organization that one would expect to be the most frequent between arabidopsis, rice and wheat, i.e. one sequence of each species (or three for the hexaploid wheat) with wheat and rice closer to each other and more distantly related to arabidopsis. Two other groups of sequences are organized in a similar way. The first group is composed of TaLTPVb.1, OsLTPV.1 and AtLTPV.1, however rice and arabidopsis are more closely related than wheat and rice. The second group is composed of AtLTPIV.1, AtLTPIV.2, OsLTPIV.3, TaLTPIVd.1 and TaLTPIVb.1. Even if a probably recent duplication in arabidopsis genome led to the presence of two copies, both are closely related to one copy of rice and two copies of wheat. In all the other cases, the arabidopsis sequences are either grouped and constitute a separated clade within a given type or branched close to the root of the type subtree. This is particularly true for AtLTPI.1, AtLTPI.4, AtLTPI.5, AtLTPI.6, AtLTPI.7, AtLTPI.8, AtLTPI.10, AtLTPI.11 and AtLTPI.12 or AtLTPIV.3, AtLTPIV.4 and AtLTPIV.5 or AtLTPVI.1, AtLTPVI.3 and AtLTPVI.4 or type II nsLTPs. In these cases, no obvious correspondence between arabidopsis and wheat/rice sequences exist and it is not possible to identify orthology relationships between nsLTP gene members of each species. A likely explanation may be that functions of nsLTPs are mostly due to a few conserved features indicating that functional domains or specific positions will be more conserved than others. Once these features are identified, it will become more relevant to perform fine phylogenetic analyses domain by domain.

The classification of the wheat nsLTP members in subfamilies when they share at least 75% amino acid identity appeared to agree with their phylogenetic relationships. Indeed, almost all the subfamilies appeared to be monophyletic (solid brackets in Figure 7) and are supported by high bootstrap values. Only two subfamilies present a more complex organization and are paraphyletic (i.e. they do not include all the members deriving from their common ancestor; dotted brackets in Figure 7). The TaLTPIIb subfamily clearly appears to be derived from TaLTPIIa. These two subfamilies share a common ancestor (node highly supported: 93), but TaLTPIIb members appear to have diverged from the others as the branch grouping them is longer and the node highly supported (98). Another subfamily, TaLTPIj, harbors surprising characteristics since the three wheat sequences are identical to three nsLTP rice copies (OsLTPI.10, OsLTPI.11 and OsLTPI.18). In contrast, we observed wheat nsLTP subfamilies (TaLTPIa, TaLTPIb, TaLTPIi, TaLTPIIa, TaLTPIIb, TaLTPIVa and TaLTPIVd indicated with green brackets in Figure 7) in which the closest related rice nsLTP is already closer to another wheat nsLTP subfamily. These wheat nsLTPs correspond either to groups in which a closer copy existed in rice and was subsequently deleted, or to wheat copies that are undergoing an evolution process specific to wheat. Because this concerns a large number of genes and the largest *TaLtpIb *subfamily, the second hypothesis is more likely.

## Discussion

Encoded by multigene families, plant nsLTPs were clustered in three clades based on their primary structure [8]. Here we report the genome-wide analysis of the *nsLtp *gene family in *O. sativa *'Nipponbare' and *A. thaliana*, which enabled us to identify six additional clades.

Gene structures and chromosomal locations indicate that the complexity of the arabidopsis and rice *nsLtp *gene families is mainly due to tandem duplication repeats representing 16 of the 49 arabidopsis *nsLtp *genes and 26 of the 52 rice *nsLtp *genes. The arabidopsis genome has undergone several rounds of genome-wide duplication events, including polyploidy [58] which likely support this *nsLtp *gene complexity. The rice genome is also the result of an ancient whole-genome duplication, a recent segmental duplication and massive ongoing individual gene duplications [59]. Characterized by Wang et al. 2005 [60], a large-scale segmental duplication is observed in rice chromosomes 11 and 12 and consists of blocks of 5.44 Mb and 4.27 Mb, respectively. Due to this genomic segmental duplication, a cluster of six tandem duplicated copies is present in both chromosomes.

Based on sequence identity, 35 rice nsLTPs and 30 arabidopsis nsLTPs are clustered in the previously described type I, type II and type III clades. Fourteen rice nsLTPs and 15 arabidopsis nsLTPs are clustered in the six new types identified in this work. In wheat, 58 out of the 122 non-redundant nsLTPs are type I nsLTPs, 29 belong to the type II and three are type III nsLTPs. Finally, 32 wheat nsLTPs were clustered in five of the new types.

The wheat EST survey failed to identify transcripts corresponding to seven genes or protein previously identified. In the case of the *TaLtpIa.2*, *TaLtpIb.1*,*TaLtpIg.1*, *TaLtpIg.5 *and *TaLtpIh.1 *genes, effective transcription is supported by isolation of cDNAs or protein. However, without cDNA or protein identified, the *TaLtpIIa.5 *and *TaLtpIb.2 *genomic sequences could be pseudogenes. In both cases, these seven haplotypes are possibly not detected in the EST databases analyzed because of inter-varietal polymorphism, or because of restricted or specific-tissue expression.

The phylogenetic tree revealed that the classification of nsLTP family members in types and subfamilies according to respectively 30% and 75% of amino acid identity enables a good representation of the organization of the family. All the types (except type II) and most of the resulting subfamilies are monophyletic and supported by convincing bootstrap values. The three species have members in all the types except arabidopsis in type VII and rice and wheat in type IX. Either type VII appeared specifically in rice/wheat lineage or has disappeared in arabidopsis. It would be interesting to trace its evolution at the monocot/dicot scale. The absence of type IX nsLTPs in rice and wheat suggests that type IX could be specific to dicot species. Search for type IX nsLTPs in other species whose whole genome was sequenced should allow confirmation of this point.

The distribution of the sequences of the three species is not homogenous. First, arabidopsis nsLTPs are grouped within types or isolated and branched close to the root of the type subtree (type II). The main conclusion we can draw from these observations is that the ancestral nsLTP gene family already included eight (or nine) types before separation between the lineage leading to arabidopsis and the lineage leading to wheat and rice, but that each type was probably represented by only one or two ancestral members. Subsequently, the family evolved specifically in each lineage in terms of copy number and speed of duplication or mutation accumulation. The alternative to this scenario would be that several copies of each type pre-existed in the ancestral nsLTP gene family before monocots and dicots diverged but that a large number of copies was lost. It would be interesting to test these hypotheses by adding nsLTPs from other species to the analysis when their complete genomic sequences become available.

Our phylogenetic approach turned out to be more informative about the evolutionary relationships of certain subfamilies, especially when based on probabilistic methods instead of computed distances. Indeed, two subfamilies (TaLTPIIa and TaLTPIj) appear to be paraphyletic, i.e. they do not include all the members derived from the same common ancestor. In the case of the TaLTPIIa subfamily, this is due to the fact that some members underwent a process of divergence which resulted in them being grouped in a different subfamily (TaLTPIIb). The TaLTPIj subfamily members appear to be grouped because they evolved not far from their closest common ancestor. Their surprisingly high level of conservation with rice nsLTPs reinforces this assumption. This subfamily groups members with common characteristics (high amino acid identity, slow evolution rates) but does not include all the descendants of the same ancestor and consequently does not represent a phylogenetic group. In conclusion, although grouping according to percentage identity may make sense, it is nevertheless important to perform a precise phylogenetic analysis to understand the relationships between the gene members. Within this context, the identification of conserved domains or residues will allow to use these specific regions to perform functional phylogenetic analysis.

Within the wheat *nsLtp *gene subfamilies for which we did not identify a closely related rice gene, it is amazing to find the largest wheat *TaLtpIb*, *TaLtpIIa*/*TaLtpIIb *gene subfamilies. The larger number of genes in these subfamilies may be the evolutionary consequence of adaptation to wheat-specific functions or various environmental changes.

Since synteny between homoeologous chromosomes was shown to be widely conserved in the hexaploid wheat *T. aestivum *[61], each gene identified should be related to two other homoeologous copies. However we report that, in single cultivars, nine *nsLtp *gene subfamilies had more than three members. In spite of the relaxed selective constraint often exerted on duplicated genes, the members of the subfamily share more than 75% identity, suggesting that recent duplications of *nsLtp *genes also occurred in the wheat genome. Diverged from a common ancestor 46 millions years ago, *Oryza *and *Triticum *species display remarkably similar genomic organization [62]. However, with more than three wheat homoeologous copies identified for most of the related rice genes, the *nsLtp *genes family appears to be much bigger in *Triticum *than in the *Oryza *genome. It has often been suggested that polyploidy offers genome plasticity, which, in turn, increases the potential ability of newly formed species to adapt to new environmental conditions [63]. When a family already presenting a high copy number at the diploid level is duplicated twice, the complexity of the redundancy and the possibilities of evolution it offers are vast. To understand the evolutionary pattern of the wheat *nsLtp *gene family, correct identification of homoeologous genes and classification of paralogous sequences is essential. To this end, gene-specific PCR primers will be designed allowing to amplify the different members of a subfamily and to determine their chromosomal locations using Chinese Spring aneuploid and deletion lines.

The high number of *nsLtp *genes in the hexaploid wheat *T. aestivum *is probably mainly due to gene duplication by polyploidization. Whether this leads to retention of function of duplicated genes or to functional diversification either at the level of gene expression or protein function remains to be determined. Depending on the species or on the gene family, both phenomena have been observed following polyploid-induced gene duplication [64].

## Conclusion

By analyzing the complete *nsLtp *gene family in both rice and arabidopsis genome we identified six new types leading to a total of nine types of nsLTPs. The type VII was found only in rice and wheat whereas the type IX was only identified in arabidopsis. Wheat EST data mining emphasized the higher number of *nsLtp *genes and complexity of certain subfamilies. The diversity of rice, arabidopsis and wheat nsLTPs suggests that nsLTPs support different functions in plants. However, until such time as specific biological functions or functional domains are defined, it seems relevant to categorize plant nsLTPs on the basis of sequence similarity and/or phylogenetic clustering.

## Methods

### *In silico *identification of rice and arabidopsis *nsLtp *genes

The Gramene rice genome database (TIGR pseudomolecule assembly release 4 of IRGSP finished sequence) [65] was searched for *nsLtp *gene sequences using the gene annotations. The TAIR arabidopsis genome database (TAIR release 6.0) [66] was searched for *nsLtp *genes annotated as encoding lipid transfer proteins and the entire arabidopsis proteome was searched for proteins displaying a HMMPfam domain PF00234 (Plant lipid transfer/seed storage/trypsin-alpha amylase inhibitor). Blastn and tblastn searches were further performed against both databases using the retrieved annotated gene sequences, the wheat *nsLtp *gene sequences and previously identified nsLTPs [32], and the wheat *nsLtp *gene sequences identified in this work. The putative rice and arabidopsis *nsLtp *gene sequences retrieved were then curated for intron-exon junction positions using the NetGen2 program [67], and from comparison with related EST sequences in the Gramene rice genome database. The amino acid sequences deduced from the newly identified rice and arabidopsis *nsLtp *genes were finally assessed through the analysis of the cysteine residue patterns.

### Wheat EST database searches

The search for *Triticum aestivum *ESTs was performed by comparing the coding sequences of wheat and rice *nsLtp *genes against EST sequences available at NCBI [68] in blastn searches. Sequence hits with *E*-values of less than 10^-4 ^and a bit score of 100 or more were identified as putative *nsLtp *homologues and extracted. EST multiple alignments were performed using the ClustalW program [69]. When their ORF alignment overlapped, multiple ESTs were considered as derived from a single gene and resolved to a single representative EST. An ORF was considered as a new gene if at least one mutation was observed and if it was represented by at least two ESTs covering the complete ORF. Then the EST displaying the most widely represented sequence in the 3'- and 5'-UTR regions was chosen as representative of the new wheat *nsLtp *gene. Singleton ESTs and ESTs presenting incomplete ORF were not considered except when several of them support a novel ORF. For a limited number of genes (11), single EST sequences displaying full ORF were nevertheless taken into account when they were supported by multiple and overlapping incomplete EST sequences.

### Amino-acid sequence analysis

Pre-proteins translated from the ORF of all nsLTP sequences were analyzed for presence of potential signal peptide cleavage sites using the SignalP 3.0 program [70]. The subcellular localization of the mature protein was predicted using the TargetP 1.1 program [71]. Following signal peptide removal, theoretical pI and MM were computed using the program provided at [72]. Amino acid sequences were efficiently aligned to the Pfam profile HMM (glocal model) defined from the protease inhibitor/seed storage/LTP family [51] using HMMalign from the HMMER package [73]. A sequence identity matrix of the mature nsLTP sequences was computed using BioEdit v7.0.4.1 [74] enabling us to determine the gene subfamily assignment and their nomenclature following the guidelines proposed by Boutrot et al. [32].

### Phylogenetic analysis

Rice, arabidopsis and wheat amino-acid sequences were aligned to the Pfam glocal model using HMMalign. Because they were not informative and created aberrant multi alignments during the re-samplings procedure, a total of 47 sites were removed from the alignment (12 of them were represented by only one sequence and the 35 others were non or few-informative sites, among them 29 were only represented by the three type VII wheat nsLTPs). Phylogenetic trees were built from the protein alignment with the maximum-likelihood method using the PHYML program [75]. Maximum-likelihood inference analyses were conducted under the Jones Taylor Thornton substitution model [76] with estimation of the proportion of invariant sites and estimation of variation rate among the remaining sites according to a gamma distribution. The confidence level of each node was estimated by the bootstrap procedure using 100 resampling repetitions of the data. The unrooted phylogenetic trees were visualized using the Treeview 1.6.6 program [77].

## Authors' contributions

FB carried out rice and wheat database searches, comparative genome analysis, gene structure prediction and nomenclature, and drafted the manuscript. NC carried out the phylogenetic analysis, contributed to the collection of the wheat EST sequences and to the writing of the manuscript. MFG coordinated the study and contributed to the writing of the manuscript. All authors read and approved the final manuscript.

## Supplementary Material

Additional file 1 Rice and arabidopsis genes encoding proteins with a Pfam domain PF00234 not identified as nsLTPs. Click here for file

Additional file 2 ***Triticum aestivum nsLtp *genes obtained from EST database analysis and features of the deduced proteins**. Identical proteins refer to their relative redundant form. Click here for file

Additional file 3 **Alignment of the rice, arabidopsis and wheat nsLTP sequences**. The mature sequences of the 122 non-redundant wheat nsLTPs, the 49 rice nsLTPs, and the 45 arabidopsis nsLTPs were aligned using HMMalign and then manually refined. The phylogenetic tree was built from this protein alignment (fasta format). Click here for file
