# Supplementary material for: Genome-wide analysis of the rice and arabidopsis non-specific lipid transfer protein (nsLtp) gene families and identification of wheat nsLtp genes by EST data mining
Source: BMC Genomics. 2008 Feb 21;9:86. doi: 10.1186/1471-2164-9-86 (PMC2277411; doi:10.1186/1471-2164-9-86)
Supplement: Additional file 1 — Rice and arabidopsis genes encoding proteins with a Pfam domain PF00234 not identified as nsLTPs. [file 1471-2164-9-86-S1.PDF]

Table 1 - Rice and arabidopsis genes encoding proteins with a Pfam domain PF00234 which belong to families other than the non-specific lipid transfer protein family.

**glycosylphosphatidylinositol-anchored proteins**	
rice	Os01g59870, Os03g07100, Os03g09230, Os03g20760, Os03g26800, Os03g26820, Os03g57970, Os03g57980, Os03g57990, Os03g58940, Os04g38840, Os05g41030, Os06g47200, Os07g07790, Os07g07870, Os07g07860, Os07g07920, Os07g07930, Os07g09970, Os07g30590, Os07g43290, Os08g42040, Os11g37320
arabidopsis	At1g03103, At1g05450, At1g18280, At1g27950, At1g36150, At1g55260, At1g62790, At1g73550, At1g73560, At1g73890, At2g13820, At2g27130, At2g44290, At2g44300, At2g48130, At2g48140, At3g22570, At3g22580, At3g22600, At3g22620, At3g43720, At3g58550, At4g08670, At4g12360, At4g14805, At4g14815, At4g22630, At4g22666, At5g09370, At5g13900, At5g64080
**hybrid proline-rich proteins**	
rice	Os02g49280, Os03g01310, Os03g14630, Os03g50960, Os03g58670, Os04g46830, Os04g52260, Os04g55170, Os06g01580, Os06g12440, Os06g43600, Os06g46780, Os06g46870, Os07g29230, Os10g09920, Os10g11310, Os10g11750, Os10g40420, Os10g40430, Os12g28880, Os12g29040
arabidopsis	At1g12090, At1g12100, At1g62500, At1g62510, At2g10940, At2g45180, At3g22120, At4g00165, At4g12470, At4g12480, At4g12490, At4g12500, At4g12510, At4g12520, At4g12530, At4g12545, At4g12550, At4g15160, At4g22460, At4g22470, At4g22490, At4g22520, At4g22610, At5g46890, At5g46900
**glycine-rich proteins**	
rice	Os10g40614
**alpha-amylase/trypsin inhibitors**	
rice	Os07g11310, Os07g11630, Os07g11650
**prolamin storage proteins**	
rice	Os06g31060, Os06g31070, Os12g16880
**2S albumin storage proteins**	
rice	Os03g46150, Os03g46180
arabidopsis	At4g27140, At4g27150, At4g27160, At4g27170, At5g54740
