# Supplementary material for: Genome-wide analysis of the rice and arabidopsis non-specific lipid transfer protein (nsLtp) gene families and identification of wheat nsLtp genes by EST data mining
Source: BMC Genomics. 2008 Feb 21;9:86. doi: 10.1186/1471-2164-9-86 (PMC2277411; doi:10.1186/1471-2164-9-86)
Supplement: Additional file 2 — Triticum aestivum nsLtp genes obtained from EST database analysis and features of the deduced proteins. Identical proteins refer to their relative redundant form. [file 1471-2164-9-86-S2.PDF]

Table S2 - *Triticum aestivum nsLtp* genes obtained from EST database analysis and features of the deduced proteins.

Identical proteins refer to their relative redundant form.

TaLtp gene nomenclature					deduced protein			
**Type** subfamily	*nsLtp* gene	former name [a]	accession number [b]	CS [c]	signal peptide	mature protein		
					AA	AA	MW	pI [d]
**Type I**								
TaLtpIa	*TaLtpIa.1*	*TaLtp9.1a*	AJ852536	CS	26	90	9607	9.86
	TaLtpIa.2	*TaLtp9.1b*	AJ784902 [e]		26	90	9609	9.95
	TaLtpIa.3		CD921562	CS	26	90	9477	8.15
TaLtpIb	*TaLtpIb.1*	*TaLtp9.2b*	AJ784901 [e]		25	90	8686	11.09
	TaLtpIb.2	*TaLtp9.2c*	AJ852555	CS	25	90	8672	11.09
	TaLtpIb.3	*TaLtp9.2d*	AJ852556	CS	25	90	8681	10.76
	TaLtpIb.4		CK218081	CS	25	90	8637	11.09
	TaLtpIb.5		CK217489	CS	25	90	8625	11.09
	TaLtpIb.6		DQ465679 [e]	CS	TaLTPIb.5			
	TaLtpIb.7		CV767212	CS	TaLTPIb.2			
	TaLtpIb.8		CV771007	CS	TaLTPIb.2			
	TaLtpIb.9		BJ209925	CS	25	90	8663	11.09
	TaLtpIb.10		BE426634	CS	25	TaLTPIb.9		
	TaLtpIb.11		CV776838	CS	25	TaLTPIb.9		
	TaLtpIb.12		CV769604	CS	25	90	8693	11.09
	TaLtpIb.13		CK166838		25	90	8705	11.09
	TaLtpIb.14		CK166935		25	90	8689	11.09
	TaLtpIb.15		CK212783	CS	25	90	8699	11.09
	TaLtpIb.16		CK170256	CS	25	90	8678	11.09
	TaLtpIb.17		AL822302		25	90	8691	11.16
	TaLtpIb.18		CV761434		25	90	8941	11.00
	TaLtpIb.19		CV766922		25	TaLTPIb.2		
	TaLtpIb.20		CV771091		25	90	8700	11.09
	TaLtpIb.21		CV774511		25	90	8727	11.09
	TaLtpIb.22		CK168001		TaLTPIb.14			
	TaLtpIb.23		CK168157		25	90	8717	11.09
	TaLtpIb.24		CJ664754	CS	TaLTPIb.15			
	TaLtpIb.25		CJ574977	CS	25	90	8729	11.09
	TaLtpIb.26		CJ702292		TaLTPIb.5			
	TaLtpIb.27		CJ664119		25	TaLTPIb.9		
	TaLtpIb.28		CJ666511		TaLTPIb.13			
	TaLtpIb.29		CJ678840	CS	25	90	8635	10.76
	TaLtpIb.30		CJ700877		TaLTPIb.16			
	TaLtpIb.31		CJ668204	CS	25	90	8651	11.09

	TaLtpIb.32		CJ559885	CS	TaLTPIb.16			
	TaLtpIb.33	*TaLtp9.5a*	AY566607	CS	25	90	8730	11.09
	TaLtpIb.34	*TaLtp9.5b*	AJ852557	CS	25	90	8804	10.85
	TaLtpIb.35		CK211307	CS	TaLTPIb.34			
	TaLtpIb.36		BE471128	CS	25	90	8774	10.85
	TaLtpIb.37		CJ671767	CS	25	90	8744	11.09
	TaLtpIb.38		CJ669479	CS	TaLTPIb.33			
	TaLtpIb.39		CJ596161		TaLTPIb.33			
TaLtpIc	*TaLtpIc.1*	*TaLtp9.3a*	AJ784899 [e]	CS	24	93	9231	10.74
	TaLtpIc.2	*TaLtp9.3b*	AJ852537		24	93	9259	10.01
	TaLtpIc.3	*TaLtp9.3c*	AJ852538		24	93	9284	10.74
	TaLtpIc.4	*TaLtp9.3d*	AJ852539	CS	24	93	9200	11.00
	TaLtpIc.5	*TaLtp9.3e*	AJ852540	CS	24	93	9268	10.74
	TaLtpIc.6	*TaLtp9.3f*	AJ852542	CS	24	91	8820	10.76
	TaLtpIc.7	*TaLtp9.3g*	AJ852543	CS	24	91	8984	10.65
	TaLtpIc.8		CD929449	CS	24	91	8977	11.00
	TaLtpIc.9		CK217646	CS	TaLTPIc.4			
	TaLtpIc.10		BM135107		24	TaLTPIc.4		
	TaLtpIc.11		CN010016		24	93	9259	10.26
	TaLtpIc.12		BF200709	CS	24	91	8895	10.76
TaLtpId	*TaLtpId.1*	*TaLtp9.4a*	AJ852541	CS	29	93	9477	10.85
	TaLtpId.2	*TaLtp9.4b*	AJ852548	CS	29	92	9466	10.70
	TaLtpId.3	*TaLtp9.4c*	AY226580 [e]	CS	29	93	9481	11.20
TaLtpIe	*TaLtpIe.1*		CV766930		29	86	8752	11.45
TaLtpIf	*TaLtpIf.1*	*TaLtp9.6a*	AJ852544		36	93	9510	11.15
	TaLtpIf.2		CA596882		36	TaLTPIf.1		
	TaLtpIf.3		CD926809		31	TaLTPIf.1		
	TaLtpIf.4		CD928098		36	TaLTPIf.1		
TaLtpIg	*TaLtpIg.1*	*TaLtp9.7a*	AJ784898 [e]		28	92	9546	10.10
	TaLtpIg.2	*TaLtp9.7b*	AJ852545	CS	28	92	9563	10.10
	TaLtpIg.3	*TaLtp9.7c*	AJ852546	CS	29	TaLTPIg.2		
	TaLtpIg.4	*TaLtp9.7d*	AJ852547	CS	28	TaLTPIg.2		
	TaLtpIg.5	*TaLtp9.7e*	AJ852559 [e]		28	92	9592	9.76
	TaLtpIg.6		BQ804678		28	TaLTPIg.2		
	TaLtpIg.7		CA700730		28	TaLTPIg.2		
	TaLtpIg.8		AW448334		27	93	9679	9.92
TaLtpIh	*TaLtpIh.1*	*TaLtp9.8a*	AAB32995 [f]		30	94	9388	11.00
	TaLtpIh.2		BQ579008	CS	33	91	9274	10.65
	TaLtpIh.3		CD927673		33	TaLTPIh.2		
	TaLtpIh.4		BQ806712	CS	30	94	9320	10.65
	TaLtpIh.5		CD900745	CS	30	94	9389	10.65

TaLtpIi	*TaLtpIi.1*		CD896537 [g]		19	92	9114	11.81
	TaLtpIi.2		CD897228 [g]		28	92	9100	11.81
TaLtpIj	*TaLtpIj.1*		CA485480 [g]	CS	25	91	8909	11.81
	TaLtpIj.2		CA485635 [g]	CS	26	92	8916	10.55
	TaLtpIj.3		CA486637	CS	25	91	8895	11.81
TaLtpIk	*TaLtpIk.1*		CA597453		27	98	9855	4.14
	TaLtpIk.2		CA596680		27	97	9798	4.14
TaLtpIl	*TaLtpIl.1*		BQ804683		37	91	9240	11.27
	TaLtpIl.2		CD890395		31	TaLTPIl.1		
	TaLtpIl.3		CA595564 [g]		34	91	9284	11.38
**Type II**								
TaLtpIIa	*TaLtpIIa.1*	*TaLtp7.1a*	AJ852549	CS	29	67	6979	9.84
	TaLtpIIa.2	*TaLtp7.1b*	AJ852550	CS	31	67	6975	9.84
	TaLtpIIa.3	*TaLtp7.1c*	AJ852554	CS	29	67	7046	9.84
	TaLtpIIa.4	*TaLtp7.1d*	AJ852551		29	67	7007	9.84
	TaLtpIIa.5	*TaLtp7.1e*	AJ852552		TaLTPIIa.4			
	TaLtpIIa.6		CD902512		23	66	7014	8.00
	TaLtpIIa.7		CD904178		28	67	7142	8.00
	TaLtpIIa.8		BE423503		29	67	7006	9.84
	TaLtpIIa.9		BQ805244	CS	29	TaLTPIIa.8		
	TaLtpIIa.10		CA731480		29	67	7019	9.84
TaLtpIIb	*TaLtpIIb.1*	*TaLtp7.2a*	AJ852553		26	67	6971	9.95
	TaLtpIIb.2		CA707197 [g]		26	67	7029	9.69
	TaLtpIIb.3		CD928801		26	68	7059	9.69
	TaLtpIIb.4		BQ161929	CS	26	67	6872	9.69
TaLtpIIc	*TaLtpIIc.1*		CK162101		28	69	7261	11.22
	TaLtpIIc.2		BQ160786	CS	29	69	7194	10.92
	TaLtpIIc.3		CA484817	CS	33	71	7437	11.16
TaLtpIId	*TaLtpIId.1*		CK211380	CS	28	68	7106	10.20
	TaLtpIId.2		CK193476	CS	28	68	7093	10.20
	TaLtpIId.3		CA643925		28	TaLTPIId.2		
	TaLtpIId.4		CD490842	CS	28	69	7143	10.31
	TaLtpIId.5		CA625596 [g]		32	69	7214	10.50
	TaLtpIId.6		CK217320	CS	28	68	7120	10.20
	TaLtpIId.7		BE442962	CS	28	68	7084	10.65
	TaLtpIId.8		BE425710	CS	28	TaLTPIId.7		
	TaLtpIId.9		CA620255 [h]		32	69	7114	10.40
TaLtpIIe	*TaLtpIIe.1*		CV760216	CS	24	67	7064	11.51
	TaLtpIIe.2		BE426770	CS	TaLTPIIe.1			
	TaLtpIIe.3		CK215937	CS	24	67	6977	10.89
	TaLtpIIe.4		CV774813	CS	24	67	6991	11.56

TaLtpIIf	*TaLtpIIf.1*		BJ256698	CS	25	70	7096	9.79
	TaLtpIIf.2		CA599453	CS	25	70	7138	9.95
TaLtpIIg	*TaLtpIIg.1*		BQ578397	CS	26	68	6930	11.74
TaLtpIIh	*TaLtpIIh.1*		CA486647	CS	16	68	6841	11.45
**Type III**								
TaLtpIIIa	*TaLtpIIIa.1*		*CA595338*	*CS*	*32*	*71*	*7107*	*9.84*
	TaLtpIIIa.2		CA727977	CS	31	71	7077	9.84
TaLtpIIIb	*TaLtpIIIb.1*		CA728517	CS	29	66	6727	10.85
**Type IV**								
TaLtpIVa	*TaLtpIVa.1*		BQ788937	CS	33	74	7726	10.10
	TaLtpIVa.2		CK215109	CS	33	74	7668	10.00
	TaLtpIVa.3		CV763900		32	74	7713	10.16
	TaLtpIVa.4		BU100298	CS	32	74	7668	9.54
	TaLtpIVa.5		BE489968	CS	30	74	7684	10.10
	TaLtpIVa.6		BQ788937	CS	33	74	7726	10.10
	TaLtpIVa.7		CJ564217	CS	30	74	7739	10.34
TaLtpIVb	*TaLtpIVb.1*		BF484978	CS	27	76	8127	4.06
	TaLtpIVb.2		BQ838625 [g]		22	TaLTPIVb.1		
	TaLtpIVb.3		CD869168	CS	35	TaLTPIVb.1		
TaLtpIVc	*TaLtpIVc.1*		CK195263	CS	28	82	8607	12.13
TaLtpIVd	*TaLtpIVd.1*		CA618855 [i]		38	80	8245	10.50
**Type V**								
TaLtpVa	*TaLtpVa.1*		CD872591	CS	24	94	9622	9.69
	TaLtpVa.2		BU100027	CS	25	93	9551	9.69
	TaLtpVa.3		BE445062	CS	18	99	9988	10.29
	TaLtpVa.4		BE444255 [g]	CS	20	99	10029	10.36
	TaLtpVa.5		BE471229	CS	20	99	9943	10.36
	TaLtpVa.6		BE444695	CS	20	99	10000	10.36
	TaLtpVa.7		BQ483156	CS	24	94	9616	9.69
	TaLtpVa.8		CD491461 [g]	CS	20	91	9240	9.86
TaLtpVb	*TaLtpVb.1*		CA732197		25	91	9397	12.20
TaLtpVc	*TaLtpVc.1*		CA594441		27	98	10514	9.69
**Type VI**								
TaLtpVIa	*TaLtpVIa.1*		CD890741	CS	19	88	9559	9.59
	TaLtpVIa.2		CD890969		19	88	9545	9.76
	TaLtpVIa.3		CD887185		19	88	9634	9.76
	TaLtpVIa.4		CD889176		19	88	9605	9.76
	TaLtpVIa.5		CJ566050	CS	19	88	9793	9.77
TaLtpVIb	*TaLtpVIb.1*		CA595166	CS	24	84	8831	4.29
	TaLtpVIb.2		CA595956		24	94	9457	4.25

	TaLtpVIb.3		CA595454	CS	24	83	8608	4.01
**Type VII**								
TaLtpVIIa	*TaLtpVIIa.1*		CA728744		24	148	15139	10.39
	TaLtpVIIa.2		CJ653476		24	150	15450	9.71
	TaLtpVIIa.3		CJ654123 [j]		24	150	15352	10.39
**Type VIII**								
TaLtpVIIIa	*TaLtpVIIIa.1*		CA700443 [g]		27	96	9482	4.59

AA, number of amino acids; CS, Chinese Spring; MM, molecular mass in Dalton; pI, isoelectric point.

[a] identified in [32]

[b] gene or representative EST GenBank accession number unless specified

[c] for the corresponding genes, at least one EST sequence presenting full homology over the ORF was identified in a cDNA library constructed using the 'Chinese Spring' cultivar

[d] cysteine residues were not taken into account in the pI calculation

[e] cDNA accession number

[f] protein accession number

[g] only one EST sequence displays the full ORF

[h] consensus sequence (in CA620255, N was replaced by G at position 382)

[i] consensus sequence (in CA618855, C was added after the nucleotide position 380, G and A were removed from the positions 308 and 386, respectively)

[j] consensus sequence (in CJ654123, N were replaced by G at position 527, G at position 544 and C at position 546)
