# Supplementary material for: Genome-wide analysis of the rice and arabidopsis non-specific lipid transfer protein (nsLtp) gene families and identification of wheat nsLtp genes by EST data mining
Source: BMC Genomics. 2008 Feb 21;9:86. doi: 10.1186/1471-2164-9-86 (PMC2277411; doi:10.1186/1471-2164-9-86)
Supplement: Additional file 3 — Alignment of the rice, arabidopsis and wheat nsLTP sequences. The mature sequences of the 122 non-redundant wheat nsLTPs, the 49 rice nsLTPs, and the 45 arabidopsis nsLTPs were aligned using HMMalign and then manually refined. The phylogenetic tree was built from this protein alignment (fasta format). [file 1471-2164-9-86-S3.DOC]

>TaLTPIa.1

-------------------IDCGHVDSL------VRP-CLSYVQGGPG-------PSGQC

CDGVKNLHNQARSQSDRQSACNCLKGIARGIH-----NLNEDNARSIPPKCGV--NLPYT

ISLNIDCSRV---------------

>TaLTPIa.2

-------------------IDCGHVDSL------VRP-CLSYVQGGPG-------PSGQC

CDGVKNLHNQARSQSDRQSACNCLRGIARGIH-----NLNEDNARGTPLKCGV--NLPYT

ISLNIDCSRV---------------

>TaLTPIa.3

-------------------IDCGHVDSL------VRP-CLSYVQGGPS-------PSGQC

CGGVQSLHNQAQSKSDRQAACNCLKGIARGIH-----NLNEDNARSLAPKCGV--NLPYH

ISLDIDCNSV---------------

>TaLTPIb.1

------------------AVSCGQVSSA------LSP-CISYARGNGAS------PSAAC

CSGVRSLASSARSTADKQAACKCIKSAAA--------GLNAGKAAGIPTKCGV--SIPYA

ISSSVDCSKIR--------------

>TaLTPIb.2

------------------AVSCGQVSSA------LSP-CISYARGNGAS------PSAAC

CSGVRSLASSARSTADKQAACKCIKSAAA--------GLNAGKAAGIPTKCGV--SVPYA

ISSSVDCSKIR--------------

>TaLTPIb.3

------------------AVSCGQVSSA------LSP-CISYARGNGAS------PSAAC

CSGVRSLASSARSTADKQAVCKCIKSAAA--------GLNAGKAAGIPTKCGV--SVPYA

ISSSVDCSKIH--------------

>TaLTPIb.4

------------------AISCGQVSSA------LSP-CISYARGNGAN------PTAAC

CSGVRSLAGAARSTADKQAACKCIKSAAG--------GLNAGKAAGIPSKCGV--SVPYA

ISASVDCSKIR--------------

>TaLTPIb.5

------------------AISCGQVSSA------LSP-CISYARGSGSS------PPAAC

CSGVRSLAGAARSTADKQAACKCIKSAAG--------GLNAGKAAGIPSKCGV--SIPYA

ISSSVDCSKIR--------------

>TaLTPIb.9

------------------AISCGQVTSA------LSP-CISYARGNGAN------PPAAC

CSGVRSLAGAARSTADKQAACKCIKSAAG--------GLNAGKAAGIPSKCGV--SVPYA

ISSSVDCSKIR--------------

>TaLTPIb.12

------------------AISCGQVTSA------LSP-CISYARGNGAN------PPAAC

CSGVRSLAGAARSTADKQAACKCIKSAAG--------GLNAGKAAGIPSKCSV--SVPYA

ISSSVDCSKIR--------------

>TaLTPIb.13

------------------AISCGQVTSA------LSP-CISYARGNVAN------PPAAC

CSGVRSLAGAARSTADKQAACKCIKSAAG--------GLNAGKAAGIPSKCGV--SVPYA

ISSSVDCSKIR--------------

>TaLTPIb.14

------------------AISCGQVTSA------LSP-CISYARGNGAN------PPAAC

CSGVRSLAGAARSTADKQAACKCIKSAAG--------GLNAGKAAGIPLKCGV--SVPYA

ISSSVDCSKIR--------------

>TaLTPIb.15

------------------AVSCGQVSSA------LSP-CISYARGNGAN------PSAAC

CSGVRSLASSARSTADKQAACKCIKSAAA--------GLNAGKAAGIPTKCGV--SVPYA

ISSSVDCSKIR--------------

>TaLTPIb.16

------------------AISCGQVTSA------LSP-CISYARGNGAN------PTAAC

CSGVRSLAGAARSTADKQAACKCIKSAAG--------GLNAGKAAGIPSKCGV--SVPYA

ISANVDCSKIR--------------

>TaLTPIb.17

------------------AISCGQVTSA------LSP-CISYARGNGAN------PTAAC

CSGVRSLAGAARSTADKQAACKCIKSAAG--------GLNAGKAAGIPSKCGV--SVPYA

ISAKVDCSKIR--------------

>TaLTPIb.18

------------------AISCGQVNSA------LSP-CISYARGNSAN------PSAAC

CSGVRRLAGAVRSTTDKKTTCNCIKSAAG--------GLSAGKAADIPSKCSV--SIPYA

ISPSLDCSTIR--------------

>TaLTPIb.20

------------------AVSCGQVSSA------LSP-CISYARGNGAS------PSVAC

CSGVRSLASSARSTADKQAACKCIKSAAA--------GLNAGKAAGIPTKCGV--SVPYA

ISSSVDCSKIR--------------

>TaLTPIb.21

------------------AVSCGQVSSA------LSP-CISYARGNGAN------PSAAC

CSGVRSLASSARSTADKQVACKCIKSAAA--------GLNAGKAAGIPTKCGV--SVPYA

ISSSVDCSKIR--------------

>TaLTPIb.23

------------------AISCGQVTSA------LSP-CISYARGNGAN------PPAAC

CSGVRSLAGAARSTADKQAVCKCIKSAAG--------GLNAGKAAGIPLKCGV--SVPYA

ISSSVDCSKIR--------------

>TaLTPIb.25

------------------AVSCGQVSSA------LSP-CISYARGNGAN------PSAAC

CSGVRSLASSARSTADKQAACKCIKSAAA--------GLNAGKAAGIPTKCGV--SVPYT

ISSSVDCSKIR--------------

>TaLTPIb.29

------------------AISCGQVTSA------LSP-CISYARGNGAN------PPAAC

CSGVRSLAGAAQSTADKQAACKCIKSAAG--------GLNAGKAAGIPSKCGV--SVPYA

ISSSVDCSKIR--------------

>TaLTPIb.31

------------------AISCGQVSSA------LSP-CISYARGNGAN------PTAAC

CSGVRSLAGAARSTADKQAACKCIKSAAG--------GLNAGKAAGIPSKCGV--SVPYA

ISATVDCSKIR--------------

>TaLTPIb.33

------------------AISCGQVNSA------LAS-CVSYAKGSGAS------PPGAC

CSGVRRLAGLARSTADKQAACRCIKSAAG--------GLNPGKAASIPSKCGV--SIPYS

ISASVDCSKIH--------------

>TaLTPIb.34

------------------AISCGQVSSA------LTP-CVAYAKGSGTS------PSGAC

CSGVRKLAGLARSTADKQATCRCLKSVAG--------GLNPNKAAGIPSKCGV--SVPYT

ISASVDCSKIH--------------

>TaLTPIb.36

------------------AISCGQVSSA------LAP-CVAYAKGSGTS------PSGAC

CSGVRKLAGLARSTADKQATCRCLKSVAG--------GLNPNKAAGIPSKCGV--SVPYT

ISASVDCSKIH--------------

>TaLTPIb.37

------------------AISCGQVNTA------LAS-CVSYAKGSGAS------PPGAC

CSGVRRLAGLARSTADKQAACRCIKSAAG--------GLNPGKAASIPSKCGV--SIPYS

ISASVDCSKIH--------------

>TaLTPIc.1

------------------AVTCSDVTSA------IAP-CMSYATGQASS------PSAGC

CSGVRTLNGKASTSADRQAACRCLKNLAGSFN-----GISMGNAANIPGKCGV--SVSFP

INNSVNCNNLH--------------

>TaLTPIc.2

------------------AVTCGDVTSA------IAP-CMSYATGQASS------PSAGC

CSGVRTLNGKASTSADRQAACRCLKNLAGSFK-----GISMGNVANIPGECGV--SVSFP

INNNVNCDTLH--------------

>TaLTPIc.3

------------------AVTCGDVMSA------IPP-CMSYATGQASS------PSAGC

CSGVRTLNGKASTSADRQAACRCLKNLAGSFN-----GISMGNAANIPGKCGV--SVSFP

INNNVNCNNLH--------------

>TaLTPIc.4

------------------AVTCGDVTSA------IAP-CMSYATGKASA------PSAGC

CSGVRTLNGKASTSADRQAACRCLKNLAGSFK-----GISMGNAATIPGKCGV--SVSFP

INTNVNCNNIH--------------

>TaLTPIc.5

------------------AVTCSDVTSA------IAP-CMSYATGQASS------PSAGC

CSGVRTLNGKASTSADRQAACRCLKNLAGSFN-----GISMGNAANIPGKCGV--SVPFP

INNNVNCNNLH--------------

>TaLTPIc.6

------------------AVTCGDVTSA------VAP-CMSYASGKASA------PSGAC

CSGVRTLNAKASTPADRKAACNCLKNLAGS-------GISMGNAASIPGKCGV--SVSIP

ISTKTNCNNLH--------------

>TaLTPIc.7

------------------AVTCGDVTSA------VAP-CMSYATGQTSA------PSAGC

CSGVRTLNAKASTSADRQAACRCLKKLAGS-------GISMGNAANIPGKCGV--SVSFP

INTKVDCNTLH--------------

>TaLTPIc.8

------------------AVTCGDVTSA------VAP-CMSYARGQASA------PSGAC

CSGVRTLNAKASTPADRKAACNCLKNLAGS-------GISMGNAANIPGKCGV--SVSFP

INTKTNCNNLH--------------

>TaLTPIc.11

------------------AVTCSDVTSA------IAP-CMSYATGQASS------PSAGC

CSGVRTLNGKASTSADRQAACRCLKNLAGSFN-----GISMGNAANIPGKCGV--SVSFP

INNNVNCDNLH--------------

>TaLTPIc.12

------------------AVTCGDVTSA------VAP-CMSYATGKASA------PSGAC

CSGVRTLNAKASTPADRKAACNCLKNLAGS-------GISMGNAANIPGKCGV--SVSFP

ISTKTNCNNLH--------------

>TaLTPId.1

------------------ALSCGQVDSK------LAP-CVAYVTGRASS------ISKEC

CSGVQGLNGMARSSSDRKIACRCLKSLATSIK-----SINMGKVSGVPGKCGV--SVPFP

ISMSTNCDTVN--------------

>TaLTPId.2

------------------ALSCGQVDSK------LAP-CVSYVTGKAPS------ISKEC

CSGVQGLNGLARSSPDRKIACRCLKSLATSIK-----SINMDKVSGVPGKCGV--SVPFP

ISMSTNCNNVN--------------

>TaLTPId.3

------------------ALSCGQVDSK------LAP-CVAYVTGRASS------ISKEC

CSGVQGLNGLARSSPDRKIACRCLKSLATSIK-----SINMGKVSGVPGKCGV--SVPFP

ISMSTNCNNVN--------------

>TaLTPIe.1

------------------ALSCSTVYNT------LMP-CLGYVQSGGV-------VPRAC

CGGIKKLVSTARSTPDRRSICTCLKNVGSGAA----GGPYVSRAAGLPGKCKV--PLPF-

-----NCNSN---------------

>TaLTPIf.1

------------------EVSCGDAVSA------LIP-CGSFLVGAVAGA-----PSESC

CRGAQGLRRMAGTPGARRALCRCLEQSGPSF------GVLPDRARQLPALCKL--GISIP

VSPHTDCDKIQ--------------

>TaLTPIg.1

-------------------ISCSTVYST------LMP-CPQYVQQGGS-------PARGC

CTGIQNLLAEANNSPDRRTICGCLKNVANGAS----GGPYITRAAALPSKCNV--ALPYK

ISPSVDCNSIH--------------

>TaLTPIg.2

-------------------ISCSTVYST------LMP-CLQYVQQGGS-------PARGC

CTGIQNLLAEANNSPDRRTICGCLKNVANGAS----GGPYITRAAALPSKCNV--ALPYK

ISPSVDCNSIH--------------

>TaLTPIg.5

-------------------ISCSTVYST------LMP-CLQYVQQGGS-------PARGC

CTGIQNLLVEANNSPDRRTICGCLENVANGAS----GGPYITRAAALPSKCNV--ALPYK

ISPSVDCNSIH--------------

>TaLTPIg.8

------------------AISCSTVYST------LMP-CLQYVQQGGS-------PARGC

CTGIQNLLAEANNSPDRRTICGCLKNVANGAS----GGPYITRADALPSKCNV--ALPYK

ISPSVDCNSNH--------------

>TaLTPIh.1

-----------------AVANCGQVVSY------LAP-CISYAMGRVSV------PGGGC

CSGVRGLNAAAATPADRKTTCTCLKQQASGMG-----GIKPNLVAGIPGKCGV--NIPYA

ISPRTDCSKVR--------------

>TaLTPIh.2

-----------------AVANCGQVVSY------LAP-CISYAMGRVSA------PGGGC

CSGVRGLNAAAATPADRKTTCTCLKQQASGIG-----GIKPNLVAGIPGKCGV--NIPYA

ISQGTDCSKVR--------------

>TaLTPIh.4

-----------------AVANCGQVVSY------LAP-CISYAMGRVSV------PGGGC

CSGVRGLNAAAATPADRKTTCTCLKQQASGMG-----GIKPNLVAGIPGKCGV--NIPYA

ISQGTDCSKVR--------------

>TaLTPIh.5

-----------------AVANCGQVVSY------LAP-CISYAMGRVSV------PGGGC

CSGVRGLNAAAATPADRKATCTCLKQQASGMG-----GIKPDLVAGIPSKCGV--NIPYA

ISPRTDCSKVR--------------

>TaLTPIi.1

------------------AISCGQVNSA------LGP-CLTYARGGAG-------PSAVC

CSGVKRLAAATQTTVDRRAVCNCLKMAVGRMS-----GFKAGNIASIPSKCGV--SVPYA

VGASVDCSRVS--------------

>TaLTPIi.2

------------------AISCGQVNSA------LGP-CLTYARGGAG-------PSAVC

CSGVKRLAAATQTTVDRRAACNCLKMAIGRMS-----GFKAGNIASIPSKCGV--SVPYA

VGASVDCSRVS--------------

>TaLTPIj.1

-------------------ITCGQVNSA------VGP-CLTYARGGAG-------PSAAC

CSGVRSLKAAASTTADRRTACNCLKNAARGIK-----GLNAGNAASIPSKCGV--SVPYT

ISASIDCSRVS--------------

>TaLTPIj.2

------------------AISCGQVNSA------VSP-CLSYARGGSG-------PSAAC

CSGVRSLNSAASTTADRRTACNCLKNVAGSIS-----GLNAGNAASIPSKCGV--SIPYT

ISPSIDCSSVN--------------

>TaLTPIj.3

-------------------ITCGQVNSA------VGP-CLTYARGGAG-------PSAAC

CSGVRSLKAAASSTADRRTACNCLKNAARGIK-----GLNAGNAASIPSKCGV--SVPYT

ISASIDCSRVS--------------

>TaLTPIk.1

------------------VVQCGQVTQL------MAP-CMPYLSGAPGMT-----PYGIC

CNSLGVLNQLAASTADRVAACNCVKAAASGGF----PAVDFSRAAALPAACGL--AINFA

VTPNMDCNQVTDEP-----------

>TaLTPIk.2

------------------VVQCGQVTQL------MAP-CMPYLSGAPGMT-----PYGIC

CNSLGVLNQLAASTADRVAACNCVKAAASGFP-----AVDFSRAAALPAACGL--AINFA

VTPNMDCNQVTDEP-----------

>TaLTPIl.1

-------------------LTCSTVYNE------LMP-CLGYVQSGGA-------VPRAC

CSGIKTLVSRARATPDRRAACACLKTVAAAAA----GGPYLGRAAGLPGRCGV--QPPFK

IDPNVNCNAV---------------

>TaLTPIl.3

-------------------LSCSTVYNE------LMP-CLGYVQSGGA-------VRRAC

CSGIKTLVSRARAKPDRRAACACLKTVAAAAA----GGPYLGRAAGLPGKCGV--QPPFK

IDPNVNCNAV---------------

>OsLTPI.1

------------------AVQCGQVMQL------MAP-CMPYLAGAPGMT-----PYGIC

CDSLGVLNRMAPAPADRVAVCNCVKDAAAGFP-----AVDFSRASALPAACGL--SISFT

IAPNMDCNQVTEELRI---------

>OsLTPI.2

------------------AISCSAVYNT------LMP-CLPYVQAGGT-------VPRAC

CGGIQSLLAAANNTPDRRTICGCLKNVANGAS----GGPYITRAAALPSKCNV--SLPYK

ISTSVNCNAIN--------------

>OsLTPI.3

-------------------VSCGDAVSA------LAP-CGPFLLGGAAR------PGDRC

CGGARALRGMAGTAEARRALCRCLEQSGPSF------GVLPDRARRLPALCKL--GLAIP

VGAATDCSKIS--------------

>OsLTPI.4

------------VVVARAALSCSTVYNT------LLP-CLPYVQSGGA-------VPAAC

CGGIRSVVAAARTTADRRAACTCLKNVAAGAA----GGPYISRAAGLPGRCGV--SVPFK

ISPNVNCNAVN--------------

>OsLTPI.5

----------------GTSDLCGLAETA------FGE-CTAYVAGGEPA------VSRRC

CRALGDIRDLAATAAERRAVCACILSEMLAAGD---GRVDSGRAAGLPAACNV--RVGFP

TSPNFNCFRVR--------------

>OsLTPI.6

--------------ADDVSVSCSDVVAD------VTP-CLGFLQGDDDH------PSGEC

CDGLSGLVAAAATTEDRQAACECLKSAVSGQF----TAVEAAPARDLPADCGL--SLPYT

FSPDVDCSQSQGHNHAFKQPNNSST

>OsLTPI.7

------------------AVTCGDVDAS------LLP-CVAYLTGKAAA------PSGDC

CAGVRHLRTLPVGTAERRFACDCVKKAAARFK-----GLNGDAIRDLPAKCAA--PLPFP

LSLDFDCNTIP--------------

>OsLTPI.8

-------------------VTCGQVVSM------LAP-CIMYATGRVSA------PTGGC

CDGVRTLNSAAATTADRQTTCACLKQQTSAMG-----GLRPDLVAGIPSKCGV--NIPYA

ISPSTDCSRVH--------------

>OsLTPI.9

------------------AVSCGDVTSS------IAP-CLSYVMGRESS------PSSSC

CSGVRTLNGKASSSADRRTACSCLKNMASSFR-----NLNMGNAASIPSKCGV--SVAFP

ISTSVDCSKIN--------------

>OsLTPI.10

-------------------ITCGQVNSA------VGP-CLTYARGGAG-------PSAAC

CSGVRSLKAAASSTADRRTACNCLKNAARGIK-----GLNAGNAASIPSKCGV--SVPYT

ISASIDCSRVS--------------

>OsLTPI.11

------------------AISCGQVNSA------VSP-CLSYARGGSG-------PSAAC

CSGVRSLNSAASTTADRRTACNCLKNVAGSIS-----GLNAGNAASIPSKCGV--SIPYT

ISPSIDCSSVN--------------

>OsLTPI.12

------------------AITCGQVGSA------IAP-CISYVTGRGG-------LTQGC

CNGVKGLNNAARTTADRQAACRCLKTLAGTIK-----SLNLGAAAGIPGKCGV--NVGFP

ISLSTDCSKVS--------------

>OsLTPI.13

------------------AITCGQVGSA------IAP-CISYVTGRSG-------LTQGC

CNGVKGLNNAARTTADRQAACRCLKSLAGSIK-----SLNLGTVAGVPGKCGV--NVGFP

ISLSTDCNKVS--------------

>OsLTPI.14

-------------------ITCGQVNSA------VGP-CLTYARGGGAG------PSAAC

CNGVRSLKSAARTTADRRTACNCLKNAARGIK-----GLNAGNAASIPSKCGV--SVPYT

ISASIDCSRVR--------------

>OsLTPI.15

-------------------VTCGQVVSM------LAP-CIMYATGRVSA------PTGGC

CDGVRTLNSAAATTADRQTTCACLKQQTSAMG-----GLRPDLVAGIPSKCGV--NIPYA

ISPSTDCSRVH--------------

>OsLTPI.16

------------------AVSCGDVTSS------IAP-CLSYVMGRESS------PSSSC

CSGVRTLNGKASSSADRRTACSCLKNMASSFR-----NLNMGNAASIPSKCGV--SVAFP

ISTSVDCSKIN--------------

>OsLTPI.17

------------------AISCGQVNSA------VSP-CLSYARGGSG-------PSAAC

CSGVRSLNSAATTTADRRTACNCLKNVAGSIS-----GLNAGNAASIPSKCGV--SIPYT

ISPSIDCSSVN--------------

>OsLTPI.18

-------------------ITCGQVNSA------VGP-CLTYARGGAG-------PSAAC

CSGVRSLKAAASTTADRRTACNCLKNAARGIK-----GLNAGNAASIPSKCGV--SVPYT

ISASIDCSRVS--------------

>OsLTPI.19

------------------AITCGQVGSA------IAP-CISYVTGRGG-------LTQGC

CNGVKGLNNAARTTADRQAACRCLKTLAGTIK-----SLNLGAAAGIPGKCGV--NVGFP

ISLSTDCSKVS--------------

>OsLTPI.20

------------------AITCGQVGSA------IAP-CISYVTGRSG-------LTQGC

CNGVKGLNNAARTTADRQAACRCLKSLAGSIK-----SLNLGTVAGVPGKCGV--NVGFP

ISLSTDCNKVS--------------

>AtLTPI.1

------------------ALSCGEVNSN------LKP-CTGYLTNGGITS-----PGPQC

CNGVRKLNGMVLTTLDRRQACRCIKNAARNVG----PGLNADRAAGIPRRCGI--KIPY-

---STQ-ISVR--------------

>AtLTPI.2

------------------LTPCEEATNL------LTP-CLRYLWAPPEAK-----PSPEC

CSGLDKVNKGVKTYDDRHDMCICLSSEAAITS------ADQYKFDNLPKLCNV--ALFAP

VGPKFDCSTIKV-------------

>AtLTPI.3

------------------AISCSVVLQD------LQP-CVSYLTSGSGN------PPETC

CDGVKSLAAATTTSADKKAACQCIKSVANSVT------VKPELAQALASNCGA--SLPVD

ASPTVDCTTVG--------------

>AtLTPI.4

----------------NALMSCGTVNGN------LAG-CIAYLTRGAP-------LTQGC

CNGVTNLKNMASTTPDRQQACRCLQSAAKAVG----PGLNTARAAGLPSACKV--NIPYK

ISASTNCNTVR--------------

>AtLTPI.5

------------------ALSCGSVNSN------LAA-CIGYVLQGGV-------IPPAC

CSGVKNLNSIAKTTPDRQQACNCIQGAARALG----SGLNAGRAAGIPKACGV--NIPYK

ISTSTNCKTVR--------------

>AtLTPI.6

------------------AVSCNTVIAD------LYP-CLSYVTQGGP-------VPTLC

CNGLTTLKSQAQTSVDRQGVCRCIKSAIGGLT---LSPRTIQNALELPSKCGV--DLPYK

FSPSTDCDSIQ--------------

>AtLTPI.7

------------------TIQCGTVTST------LAQ-CLTYLTNSGP-------LPSQC

CVGVKSLYQLAQTTPDRKQVCECLKLAGKEIK-----GLNTDLVAALPTTCGV--SIPYP

ISFSTNCDSISTAV-----------

>AtLTPI.8

------------------AISCGAVTGS------LGQ-CYNYLTRGGF-------IPRGC

CSGVQRLNSLARTTRDRQQACRCIQGAARALG----SRLNAGRAARLPGACRV--RISYP

ISARTNCNTVR--------------

>AtLTPI.9

-------------------IACPQVNMY------LAQ-CLPYLKAGGN-------PSPMC

CNGLNSLKAAAPEKADRQVACNCLKSVANTIP-----GINDDFAKQLPAKCGV--NIGVP

FSKTVDCNSIN--------------

>AtLTPI.10

------------------AISCNAVQAN------LYP-CVVYVVQGGA-------IPYSC

CNGIRMLSKQATSASDKQGVCRCIKSVVGRVSY---SSIYLKKAAALPGKCGV--KLPYK

IDPSTNCNSIK--------------

>AtLTPI.11

------------------AITCGTVASS------LSP-CLGYLSKGGV-------VPPPC

CAGVKKLNGMAQTTPDRQQACRCLQSAAK--------GVNPSLASGLPGKCGV--SIPYP

ISTSTNCATIK--------------

>AtLTPI.12

------------------AISCGTVAGS------LAP-CATYLSKGGL-------VPPSC

CAGVKTLNSMAKTTPDRQQACRCIQSTAKSIS-----GLNPSLASGLPGKCGV--SIPYP

ISMSTNCNNIK--------------

>TaLTPIIa.1

--------------------ACQ--ASQ------LAV-CASAILSGAK-------PSGEC

CGNLRA-----------QQGCFCQYAKDPTYG----QYIRSPHARDTLTSCGL--AVPH-

------C------------------

>TaLTPIIa.2

--------------------ACQ--ASQ------LAV-CASAILSGAK-------PSGEC

CGNLRA-----------QQGCFCQYAKDPNYG----QYIRSPHARDTLHSCGL--AVPH-

------C------------------

>TaLTPIIa.3

--------------------ACQ--ASQ------LAV-CASAILSGAK-------PSGEC

CGNLRA-----------QQPCFCQYAKDPTYG----QYIRSPHARDTLQSCGL--AVPH-

------C------------------

>TaLTPIIa.4

--------------------ACR--ASQ------LAV-CASAILSGAK-------PSGEC

CGNLRA-----------QQGCFCQYAKDPTYG----QYIRSPHARDTLTSCGL--AVPH-

------C------------------

>TaLTPIIa.6

---------------------CE--VTQ------LAV-CASAILGGTK-------PSGEC

CGNLRA-----------QQGCFCQYVKDPNYG----HYVNSPHARETLQTCGI--ALPH-

------C------------------

>TaLTPIIa.7

--------------------ECQ--VTQ------LAV-CASAILGGTK-------PSGEC

CGNLRA-----------QQGCFCQYVKDPNYG----HYVNSPHARETLQTCGI--ALPH-

------C------------------

>TaLTPIIa.8

--------------------ACQ--ASQ------LAV-CASAILSGAK-------PSGEC

CGNLRA-----------QQGCFCQYAKDPTYG----QYIRSPHARDTLQSCGL--AVPH-

------C------------------

>TaLTPIIa.10

--------------------ACQ--ASQ------LAV-CASAILSGAK-------PSGEC

CGNLRA-----------QQPCFCQYAKDPTYG----QYIRSPHARDTLTSCGL--AVPH-

------C------------------

>TaLTPIIb.1

--------------------ACE--VGQ------LTV-CMPAITTGAK-------PSGAC

CGNLRA-----------QQACFCQYAKDPSLA----RYITSPHARETLVSCGL--AVPH-

------C------------------

>TaLTPIIb.2

--------------------ACE--VGQ------LTV-CMPAITTGAK-------PSDAC

CGNLRA-----------QQACFCQYAKDPSLA----RYITSPHARETLVSCGL--AVPH-

------C------------------

>TaLTPIIb.3

--------------------ACE--VGQ------LTV-CMPAITTGAK-------PSEAC

CGSLRA-----------QQACFCQYAKDPSLG----AYIRSPHARETLVSCGL--AVPH-

------CS-----------------

>TaLTPIIb.4

--------------------ACE--VGQ------LTV-CMPAITTGAK-------PSGAC

CANLRA-----------QQACFCQYAKDPSLG----AYIRSPHASETLVSCGL--AVPH-

------C------------------

>TaLTPIIc.1

-------------------ATCS--PTQ------LTP-CAPAIIGNAA-------PSAAC

CGKLKAH----------PASCLCKYKKDPNLQ----RYVNSPNGKKVFAACKL--RLPR-

------C------------------

>TaLTPIIc.2

-------------------ATCS--PTQ------LTP-CAPAIIGNAA-------PSAAC

CGKLKAH----------PASCLCKYKKDPNLQ----RYVNSPSGKKVFTACKL--RLPS-

------C------------------

>TaLTPIIc.3

-----------------ATTACV--PTQ------LTP-CAPAIVGNAA-------PTAAC

CARLKAH----------PASCFCQYKKNPNMQ----RYVNSPNGKKVFAACKV--PLPK-

------C------------------

>TaLTPIId.1

-------------------ATCN--ALQ------LTP-CAGAIVGNAA-------PTASC

CSKMKE-----------QQPCMCQYARDPNLK----QYVDSPNGKKVMAACKV--PVPS-

------C------------------

>TaLTPIId.2

-------------------ATCN--ALQ------LTP-CAGAIIGSAA-------PTASC

CSKMKE-----------QQPCMCQYARDPNLK----QYVDSPNGKKVMAACKV--PVPS-

------C------------------

>TaLTPIId.4

------------------ATACD--ATQ------LTP-CAGAIIGSSP-------PTAAC

CSRLKE-----------QQPCLCTYARDPNLQ----RYVNSPNGKKAMAACKV--PVPS-

------C------------------

>TaLTPIId.5

------------------ATACD--ATQ------LTP-CAGAIIIGRS-------PSAAC

CSRLKE-----------QQPCLCTYARDPNLQ----RYVNSPNGKKAMAACKV--PVPS-

------C------------------

>TaLTPIId.6

-------------------ATCN--ALQ------LTP-CAGAIIGNAA-------PTASC

CSKMKE-----------QQPCMCQYARDPNLK----QYVDSPNGKKVMAACKV--PVPS-

------C------------------

>TaLTPIId.7

-------------------ATCD--ALQ------LSP-CAGAIVGNAA-------PTAVC

CSRMKA-----------QRPCMCQYARDPNLK----QYVNSPNGKKVLAACKV--PVPS-

------C------------------

>TaLTPIId.9

------------------ATACD--AAQ------LTP-CAGAIIGSSP-------PTAAC

CSRLRE-----------QQPCLCTYARDPKLQ----RYVSSPNGKNAMAACKV--PVPS-

------C------------------

>TaLTPIIe.1

--------------------QCN--AGN------LAV-CASPIVSGTP-------PSKTC

CNNLKS-----------QRGCFCQFAHNRAYS----SYINSPNARKTLVSCGV--PVPK-

------C------------------

>TaLTPIIe.3

--------------------QCN--AGS------LAV-CASPIISGAK-------PSTTC

CNNLKS-----------QRGCFCQYARNPAYS----SYINSPNARKTLTSCGV--AVPK-

------C------------------

>TaLTPIIe.4

--------------------QCN--AGS------LAV-CTSPIISGTP-------PSKTC

CNNLKS-----------QRGCFCKFARNPAYS----SYINSRNAGKTLTSCGI--AVPK-

------C------------------

>TaLTPIIf.1

-------------------QDCD--AGK------LIV-CAAAIIGGAE-------PSASC

CSNLKA-----------QQGCLCKYASNPAYS----GYINSPTARKTLTSCGI--PIPT-

------CPQ----------------

>TaLTPIIf.2

-------------------QDCD--AGK------LIV-CAAAIIGGAE-------PSASC

CTNLKA-----------QRGCLCKYASNPAYS----GYINSPTTRKTLASCGI--PIPT-

------CPQ----------------

>TaLTPIIg.1

-------------------AGCD--ASA------LRP-CVGAIMLGGA-------VTPGC

CARLRA-----------QRACLCQYARDPSYR----GYVNSPRAQSVVAACGL--PGPK-

------C------------------

>TaLTPIIh.1

-------------------ASCN--AGQ------LTV-CASAMLSGAA-------PSAAC

CSNLKA-----------QQGCLCQFAKNPAYA----RYVNSPNARKTVASCGV--ALPR-

------C------------------

>OsLTPII.1

----------ASRTAPAAATKCD--PLA------LRP-CAAAILWGEA-------PSTAC

CAGLRA-----------QKRCLCRYAKNPDLR----KYINSQNSRKVAAACSV--PAPR-

------C------------------

>OsLTPII.2

--------------RASKKASCD--LMQ------LSP-CVSAFSGVGQ-----GSPSSAC

CSKLKAQ----------GSSCLCLYKDDPKVK----RIVSSNRTKRVFTACKV--PAPN-

------C------------------

>OsLTPII.3

------------GVVGVAGAGCN--AGQ------LTV-CTGAIAGGAR-------PTAAC

CSSLRA-----------QQGCFCQFAKDPRYG----RYVNSPNARKAVSSCGI--ALPT-

------CH-----------------

>OsLTPII.4

--------------------ACD--ALQ------LSP-CASAIIGNAS-------PSASC

CSRMKE-----------QQPCLCQYARDPNLQ----RYVNSPNGKKVLAACHV--PVPS-

------C------------------

>OsLTPII.5

-------------------ATCT--PTQ------LTP-CAPAIVGNSP-------PTAAC

CGKLKAH----------PASCFCQYKKDPNMK----KYVNSPNGKKVFATCKV--PLPK-

------C------------------

>OsLTPII.6

-------------------AGCN--PSA------LSP-CMSAIMLGAA-------PSPGC

CVQLRA-----------QQPCLCQYARDPSYR----SYVTSPSAQRAVKACNV--KAN--

------C------------------

>OsLTPII.7

--------------QAPPPPQCD--PGL------LSP-CAAPIFFGTA-------PSASC

CSSLKA-----------QQGCFCQYAKDPTYA----SYINSTNARKMIAACGI--PLPN-

------CG-----------------

>OsLTPII.8

--------------QSPPPPQCD--PGL------LSP-CAAPIFFGTA-------PSASC

CSSLKA-----------QQGCFCQYAKDPMYA----SYINSTNARKMIAACGI--PLPN-

------CG-----------------

>OsLTPII.9

-------------QAPPPPPQCD--PGL------LSP-CAAPIFFGTA-------PSASC

CSSLKA-----------QQGCFCQYAKDPTYA----SYINSTNARKMIAACGI--PFPN-

------CS-----------------

>OsLTPII.10

--------------QAPPPVQCD--PGK------LSA-CAVPIFFGTA-------PSKSC

CSNLRAQE---------KDGCFCQYARDPMYA----SYINSTNARNTIAACGI--AFPS-

------C------------------

>OsLTPII.11

--------------------QCD--PEQ------LSA-CVSPIFYGTA-------PSESC

CSNLRAQQ---------KEGCLCQYAKDPTYA----SYVNNTNARKTIAACGI--PIPS-

------C------------------

>OsLTPII.12

--------------------QCN--AGQ------LAI-CAGAIIGGST-------PSASC

CSNLRA-----------QRGCFCQYARNPAYA----SYINSANARKTLTSCGI--AIPR-

------C------------------

>OsLTPII.13

--------------AVVPPSRCN--PTL------LTP-CAGPALFGGP-------VPPAC

CAQLRA-----------QAACLCAYARSPNYG----SYIRSPNARRLFAVCGL--PMPQ-

------CS-----------------

>AtLTPII.1

----------LRVLSEDKKVACI--VTD------LQV-CLSALETPIP-------PSAEC

CKNLKI-----------QKSCLCDYMENPSIE----KYL--EPARKVFAACGM--PYPR-

------C------------------

>AtLTPII.2

---------KTLILGEEVKATCD--FTK------FQV-CKPEIITGSP-------PSEEC

CEKLKE-----------QQSCLCAYLISPSIS----QYI--GNAKRVIRACGI--PFPN-

------CS-----------------

>AtLTPII.3

--------GIVKVSWGEKKVACT--VTE------LQP-CLPSVIDGSQ-------PSTQC

CEKLKE-----------QNSCFCDYLQNPQFS----QYI--TAAKQILAACKI--PYPN-

------C------------------

>AtLTPII.4

-------------------VTCS--PMQ------LAS-CAAAMTSSSP-------PSEAC

CTKLRE-----------QQPCLCGYMRNPTLR----QYVSSPNARKVSNSCKI--PSPS-

------C------------------

>AtLTPII.5

---------EDTGDTGNVGVTCD--ARQ------LQP-CLAAITGGGQ-------PSGAC

CAKLTE-----------QQSCLCGFAKNPAFA----QYISSPNARKVLLACNV--AYPT-

------C------------------

>AtLTPII.6

------------------VDPCN--PAQ------LSP-CLETIMKGSE-------PSDLC

CSKVKE-----------QQHCICQYLKNPNFK----SFLNSPNAKIIATDCHC--PYPK-

------C------------------

>AtLTPII.7

---------VVVRVEEEEKVVCI--VTD------LRV-CLPAVEAGSQ-------PSVQC

CGKLKE-----------QLSCLCGYLKIPSFT----QYVSSGKAQKVLTACAI--PIPK-

------C------------------

>AtLTPII.8

---------------DEMMGRC---MHE------IAN-CLVAIDKGTK-------LPSYC

CGRMVK-----------PQPCACKYFIKNPVL-----------LPRLLIACRV--PHPK-

------C------------------

>AtLTPII.9

-------------------VTCS--PMQ------LSP-CATAITSSSP-------PSALC

CAKLKE-----------QRPCLCGYMRNPSLR----RFVSTPNARKVSKSCKL--PIPR-

------C------------------

>AtLTPII.10

-----------------VNQACN--KIE------ITG-CVPAILYGDK-------PTTQC

CEKMKA-----------QEPCFFYFIKNPVFN----KYVTSPQARAILKCCGI--PYPT-

------C------------------

>AtLTPII.11

--------TVVGGWGIEEKAACI--VTN------LMS-CLPAILKGSQ-------PPAYC

CEMLKE-----------QQSCLCGYIKSPTFG----HYVIPQNAHKLLAACGI--LYPK-

------C------------------

>AtLTPII.12

--------RVVKGSGEEVNVTCD--ATQ------LSS-CVTAVSTGAP-------PSTDC

CGKLKE-----------HETCLCTYIQNPLYS----SYVTSPNARKTLAACDV--AYPT-

------C------------------

>AtLTPII.13

--------TEVKLSGGEADVTCD--AVQ------LSS-CATPMLTGVP-------PSTEC

CGKLKE-----------QQPCFCTYIKDPRYS----QYVGSANAKKTLATCGV--PYPT-

------C------------------

>AtLTPII.14

----------------EETQSCV--PME------LMP-CLPAMTKREQ-------PTKDC

CENLIK-----------QKTCLCDYIKNPLYS----MFTISLVARKVLETCNV--PYTS-

------C------------------

>AtLTPII.15

----------------EVSSSCI--PTE------LMP-CLPAMTTGGQ-------PTKDC

CDKLIE-----------QKECLCGYINNPLYS----TFVSSPVARKVLEVCNI--PYPS-

------C------------------

>TaLTPIIIa.1

---------------QPPLGTCGAQLSQ------LAP-CARYSVPPLP-GQALPTPGPEC

CSALGSV----------SRDCACGAID---------------IINSLPAKCGL--PRVS-

------CQ-----------------

>TaLTPIIIa.2

---------------QPPLGTCGAQLSQ------LAP-CARYSVPPLP-GQALPAPGPEC

CSALGSV----------SRDCACGAID---------------IINSLPAKCGL--PRVS-

------CQ-----------------

>TaLTPIIIb.1

----------------QPPGGCVPQLNR------LLA-CRAYLVPGAA------DPSADC

CSALSSI----------SRDCACSTMG---------------IINSIPSRCNI--GRVN-

------CSA----------------

>OsLTPIII.1

--------------QGGGGGECVPQLNR------LLA-CRAYAVPGAG------DPSAEC

CSALSSI----------SQGCACSAIS---------------IMNSLPSRCHL--SQIN-

------CSA----------------

>OsLTPIII.2

-----------------QQPSCAAQLTQ------LAP-CARVGVAPAP-GQPLPAPPAEC

CSALGAV----------SHDCACGTLD---------------IINSLPAKCGL--PRVT-

------CQ-----------------

>AtLTPIII.1

-------------------QQCRDELSN------VQV-CAPLLLPGAV----NPAANSNC

CAALQAT----------NKDCLCNALR---------------AATTLTSLCNL--PSFD-

------CGISA--------------

>AtLTPIII.2

-------------------QSCNAQLST------LNV-CGEFVVPGAD---RTN-PSAEC

CNALEAV----------PNECLCNTFR---------------IASRLPSRCNI--PTLS-

------CS-----------------

>AtLTPIII.3

-------------------QECGNDLAN------VQV-CAAMVLPGSG------RPNSEC

CAALQST----------NRDCLCNALR---------------AATSLPSLCNL--PPVD-

------CGINA--------------

>TaLTPIVa.1

------------------IHVCNVDTGS-----MLNN-CRSYCSVGSN----EASPSGAC

CGAVRGA----------NFKCLCKYKGFLPK------DIDANRAMQIPAKCGY--GPAS-

------C------------------

>TaLTPIVa.2

------------------YHVCNVDTDS-----LVNN-CRSYCAVGSN----EASPSGAC

CGAVRGA----------NFKCLCKYKGLLPK------GIDANRAMQIPAKCGY--GAAS-

------C------------------

>TaLTPIVa.3

------------------LRVCSVDRDS-----VVNN-CKSYCTVGST----EASPSGAC

CNAVRGG----------NFKCLCQFRNALSP------DIDGNRAMQIPAKCGY--GAAS-

------C------------------

>TaLTPIVa.4

------------------YHVCNVDTDS-----LVNN-CKSYCTAGSS----EASPSGAC

CGAVRGG----------DFHCLCQYKGVLPK------NIDANRAMQIPGKCGY--GAVS-

------C------------------

>TaLTPIVa.5

------------------IHVCNVDTGS-----MVNN-CRSYCTVGSN----EASPSGAC

CGAVRGA----------NFKCLCKYKGLLSK------DIDANRVMQIPAKCGY--GAAS-

------C------------------

>TaLTPIVa.6

------------------IHVCNVDTGS-----MLNN-CRSYCSVGSN----EASPSGAC

CGAVRGA----------NFKCLCKYKGFLPK------DIDANRAMQIPAKCGY--GPAS-

------C------------------

>TaLTPIVa.7

------------------IHVCNVDTGS-----MVNN-CRSYCRVGSN----EASPSGAC

CGAVRGA----------NFKCLCKYKGLLSK------DIDANRVMQIPAKCGY--GAAS-

------C------------------

>TaLTPIVb.1

--------------------VCDMDNDD------FMA-CQPAAAATTD---PQPAPSEAC

CATLGKA----------DLRCLCSYKNSPWLS---LYNIDPKRAMELPAKCGL--TTPP-

-----DC------------------

>TaLTPIVc.1

--------------APGPLMMCNVDVYR-----MIGA-CRSYCARGSR----EATPSGQC

CAALRGA----------NLRCVCQKKGLLASA----GNIDARRAMQIPSKCGI-GNVPS-

-----RC------------------

>TaLTPIVd.1

-------------------AVCDMSNEQ------FMS-CQPAAAKTTD---PPAAPSQAC

CDALGGG----------GPQVPVRLQELAVDG---RLQHRPQARHGTSGKCGS-PRRPT-

---ATVC------------------

>OsLTPIV.1

--------------AGAPFMVCGVDADR-----MAAD-CGSYCRAGSR----ERAPRREC

CDAVRGA----------DFKCLCKYRDELRVM----GNIDAARAMQIPSKCRIKGAPKS-

------C------------------

>OsLTPIV.2

------------------LSMCGVDRSA------VAL-CRSYCTVGSA----EKAPTKEC

CKAVANA----------DFQCLCDRRDMLRNL----ENIDADRATQIPSKCGVPGASSS-

------CK-----------------

>OsLTPIV.3

--------------------VCNMSNDE------FMK-CQPAAAATSN---PTTNPSAGC

CSALSHA----------DLNCLCSYKNSPWLSIY---NIDPNRAMQLPAKCGL--TMPA-

-----NC------------------

>OsLTPIV.4

------------------HGICNLSDAG------LQA-CKPAAAVRNP----ADTPSSEC

CDALAAA----------DLPCLCRYKGSAGAR--RFYGIDLNRAMTLPGKCGL--TLPA-

-----HC------------------

>AtLTPIV.1

------------------IDLCGMSQDE------LNE-CKPAVSKENP-----TSPSQPC

CTALQHA----------DFACLCGYKNSPWLGS---FGVDPELASALPKQCGL-ANAPT-

------C------------------

>AtLTPIV.2

------------------IDLCGMTQAE------LNE-CLPAVSKNNP-----TSPSLLC

CNALKHA----------DYTCLCGYKNSPWLGS---FGVDPKLASSLPKECDL-TNAPT-

------C------------------

>AtLTPIV.3

------------------MSICDMDIND------MQK-CRPAITGNNP-----PPPVNDC

CVVVRKA----------NFECLCRFKFYLPIL-----RIDPSKVVALVAKCGV--TTVP-

----RSCQV----------------

>AtLTPIV.4

------------------IPVCNIDTND------LAK-CRPAVTGNNP-----PPPGPDC

CAVARVA----------NLQCLCPYKPYLPTV-----GIDPSRVRPLLANCGV--NSPS-

------CF-----------------

>AtLTPIV.5

---------------------CNINANH------LEK-CRPAVIGDNP-----PSPIKEC

CELLQAA----------NLKCICRFKSVLPV-----LAVYPSKVQALLSKCGLTTIPPA-

------CQALRN-------------

>TaLTPVa.1

---------------AEGAGECGRSSPDRMALR-MAP-CISAADEPDS------APSSSC

CSAVHTIGK--------SPSCLCAVMLSGTAKM---AGIKPEVAITIPKRCNM-ADRPV-

---GYKCGDYTLP------------

>TaLTPVa.2

----------------EGAGECGRSSPDRMALR-MAP-CISAADEPDS------APSSSC

CSAVHTLGK--------SPSCLCAVMLSGTAKM---AGIKPEVAITIPKRCNM-ADRPV-

---GYKCGDYTLP------------

>TaLTPVa.3

-----------APRGAHGAGECGRTPADEMALK-LAP-CASAGQDPNS------APSSGC

CTAVRTIGK-------QSPKCLCAVMLSDTAKS---AGIKPEAAISIPKRCNL-VDRPV-

---GYKCGAYTLP------------

>TaLTPVa.4

-----------APRGAHGAGECGKTPADKMALK-LAP-CASAGQDPKS------VPSSGC

CTAVHTIGK-------QSPKCLCAVMLSDTAKS---AGIKPEVAMSIPKRCNL-VDRPV-

---GYKCGAYTLP------------

>TaLTPVa.5

-----------APRGAHGAGECGKTPADKMALK-LAP-CASAGQDPKS------APSSGC

CAAVHTIGK-------QSPKCLCAVMLSDTAKS---AGIKPEAAMSIPKRCNL-VDRPV-

---GYKCGAYTLP------------

>TaLTPVa.6

-----------APRGAHGAGECGKTPADKMALK-LAP-CASAGQDPKS------APSSGC

CTAVHTIGK-------QSPKCLCAVMLSDTAKS---AGIKPEVAMSIPKRCNL-VDRPV-

---GYKCGAYTLP------------

>TaLTPVa.7

---------------AEGAGECGRASPDRMALR-MAP-CISAADEPDS------APSSSC

CSAVHTIGK--------SPSCLCAVMLSGTAKM---AGIRPEVAITIPKRCNI-ADRPV-

---GYKCGDYTLP------------

>TaLTPVa.8

------------------AGECGRASADRVALR-LAP-CVSAADDPQS------TPTSSC

CSAVHAIG--------QSPSCLCAVMLSGTARA---AGIKPEVAITIPKRCNM-ADRPV-

---GYKCGDYTLP------------

>TaLTPVb.1

------------------AGECGRVPADRMALK-LAP-CAAATQNPRA------KVAPGC

CAQIRSIGR--------SPKCLCAVMLSSTARQ---AGVKPAVAMTIPKRCAL-ANRPI-

---GYKCGPYTLP------------

>TaLTPVc.1

------------------KDECGATPPDQEALK-LVP-CVAAGKDPDS------KPSDRC

CAAVKEIGER-------SPACLCAVLLSKIVRR---VGVKPEVAITIPKRCDL-TDRPI-

---GYKCGDYTMPSLQLKD------

>OsLTPV.1

------------------AGECGRVPVDQVALK-LAP-CAAATQNPRA------AVPPNC

CAQVRSIGR--------NPKCLCAVMLSNTARS---AGVKPAVAMTIPKRCAI-ANRPI-

---GYKCGPYTLP------------

>OsLTPV.2

----------------DGAGECGATPPDKMALK-LAP-CASAAKDPKS------TPSSGC

CTAVHTIGK-------QSPKCLCAVMLSSTTR---NAGIKPEVAITIPKRCNI-ADRPV-

---GYKCGDYTLP------------

>OsLTPV.3

------------------AGKCGKTPAEKVALK-LAP-CAKAAQDPGA------RPPAAC

CAAVRDIGT------HQSHACLCAVLLSSTVRR---SGVKPEVAITIPKRCKL-ANRPV-

---GYKCGAYTLPSLQG--------

>OsLTPV.4

----------------EGAGECGRASADRVALR-LAP-CVSAADDPQS------APSSSC

CSAVHTIG--------QSPSCLCAVMLSNTARV---AGIKPEVAITIPKRCNM-ADRPV-

---GYKCGDYTLP------------

>AtLTPV.1

------------------AGECGRMPINQAAAS-LSP-CLPATKNPRG------KVPPVC

CAKVGALIR-------TNPRCLCAVMLSPLAKK---AGINPGIAIGVPKRCNI-RNRPA-

---GKRCGRYIVP------------

>AtLTPV.2

------------------AGECGRSSPDNEAMK-LAP-CAGAAQDANS------AVPGGC

CTQIKRFS--------QNPKCLCAILLSDTAKA---SGVDPEVALTIPKRCNF-ANRPV-

---GYKCGAYTLP------------

>AtLTPV.3

------------------AGECGRNPPDREAIK-LAP-CAMAAQDTSA------KVSAIC

CARVKQMG--------QNPKCLCAVMLSSTARS---SGAKPEISMTIPKRCNI-ANRPV-

---GYKCGAYTLP------------

>TaLTPVIa.1

-------------------EDCVVDLKG-----IIRE-CKPYVMFPASPKI---TPASAC

CSVVQKV----------NAPCMCSKVTKEIEK-----VVCMDKVVYVADYCKN--PLKP-

---GSDCGSYHVPSQGQ--------

>TaLTPVIa.2

-------------------EDCTVDLKG-----LIRE-CKPYVVFPASPKI---TPSSAC

CSAVQKV----------NAPCMCSKVTKEIEK-----VVCMDKVVYVADYCKN--PLKP-

---GSDCGSYHVPSQGR--------

>TaLTPVIa.3

-------------------EDCTVDLKG-----LIRE-CKQYVMFPASPKI---TPSSAC

CSVVQKV----------NSPCLCSKVTKEIEK-----VVCMDKVVYVADYCKN--PLKP-

---GSDCGSYHVPSQGR--------

>TaLTPVIa.4

-------------------EDCTVDLKG-----LIRE-CKPYVMFPASPKI---TPSSAC

CSVVQKV----------NAPCMCSKVTKEIEK-----VVCMDKVVYVADYCKN--PLKP-

---GSDCGSYHVPSQGR--------

>TaLTPVIa.5

-------------------EDCTVDLKG-----LIRE-CKQYVMFPASPKI---TPSSAC

CSVVQKV----------NSPCLCSKVTKEIEK-----VVCMDKVVYVADYYKN--PLKP-

---GSDCGSYHVPSQRR--------

>TaLTPVIb.1

----------------EGPAGCQDDVVA------LNEACYQYVQKGAP---TVP-PSQEC

CDAVRRV----------DVPCVCSYLGSPGVRD----NISMEKVFYVSQQCGV--SIPG-

-----NCGGSKV-------------

>TaLTPVIb.2

------AGPAAGGAAVADPAGCQDDVVA------LNEACYKYVQKGAP---TVP-PSQEC

CDAVKSV----------DVPCVCSYLGSPGVRD----NISMEKVFYVSQQCGV--SIPG-

-----NCGGSKV-------------

>TaLTPVIb.3

-----------------GPAGCQDDVVA------LNEACYQYVQKGAP---TVP-PSQEC

CDAVKSV----------DVPCVCSYLGSPGVRD----NISMEKVFYVSQQCGV--SIPG-

-----NCGGSMV-------------

>OsLTPVI.1

----ARPATSSTADAPATSGDCSSDVQD-----LMAN-CQDYVMFPADPKI---DPSQAC

CAAVQRA----------NMPCVCNKVIPEVEQ-----LICMDKVVYVVAFCKK--PFQP-

---GSNCGSYRVPASLA--------

>OsLTPVI.2

------------------DEGCSRDLQD-----LIME-CQKYVMNPANPKI---EPSNAC

CSVIQKA----------NVPCLCSKVTKEIEK-----IVCMEKVVYVADYCKK--PLQP-

---GSKCGSYTIPSLQQ--------

>OsLTPVI.3

-------------------TECQNDVEV-----LKTT-CYKFV-EKDGPKL---QPSPDC

CTSMKGV----------NVPCVCTYLGSPGVRD----NINMDKVFYVTKQCGI--AIPG-

-----NCGGSKV-------------

>OsLTPVI.4

-----------ATVSPSAADKCEKDLDL-----LMGS-CEGYLRFPAEAKA---APSRAC

CGAVRRV----------DVGCLCGMVTPEVEQ-----YVCMDKAVYVAAYCHR--PLLP-

---GSYCGSYHVPGPVV--------

>AtLTPVI.1

----------------DLRKGCYDLGIT-----VLMG-CPDSIDKKLPAPP---TPSEGC

CTLVRTI----------GMKCVCEIVNKKIED-----TIDMQKLVNVAAACGR--PLAP-

---GSQCGSYRVPGA----------

>AtLTPVI.2

---------------VPGQGTCQGDIEG-----LMKE-CAVYVQRPGP-KV---NPSEAC

CRVVKRS----------DIPCACGRITASVQQ-----MIDMDKVVHVTAFCGK--PLAH-

---GTKCGSYVVP------------

>AtLTPVI.3

-------------------QVCGANLSG-----LMNE-CQRYVSNAGP--NSQP-PSRSC

CALIRPI----------DVPCACRYVSRDVTN-----YIDMDKVVYVARSCGK--KIPS-

---GYKCGSYTIPAA----------

>AtLTPVI.4

-------------------ERCNDSGIE-----VLRG-CPDSI-DKELPTP--PRPSQGC

CTLVRII----------GMECVCEVINKEIEA-----AIDMQKLVNVAAACGR--PLAP-

---GSQCGSYLVPGGMIRH------

>TaLTPVIIa.1

KDIIASEPTIPRPAAVDVSATCMGSLLE------LSP-CLAFFRD-----AGTSKAPAGC

CKGLGTIVR-------DQPACLCHIFNHTLERAI---IPVNRALALIRDVCGL--TPPK-

---VASCANAGAVPPLYVCPAPSA-

>TaLTPVIIa.2

KHIIASEPTIPRPSAVDVSATCMGSLLE------LSP-CLAFFRD-----AGTSKAPAGC

CKGLGTIVR-------DQPACLCHIFNRTLERAI---IPVDRALALMGDVCGL--TLPQ-

-DLMSSCGDNGGVPPLYVCPAPSA-

>TaLTPVIIa.3

KDIIASEPTIPRPAAVDVSATCMGSLLE------LSP-CLAFFRD-----AGTSKAPAGC

CKGLGSIVR-------DQPACLCHIFNHTLERAI---IPVNRALALIRDVCGL--TPPK-

-NLMASCANGGAVPPLYVCPAPSA-

>OsLTPVII.1

-----------------AATTCVASLLE------LSP-CLPFFKDKAA-----TAAPEGC

CAGLSSIVK-------GEAVCLCHIVNHTLERAI---IPVDRAFALLRDVCRL--SPPA-

-DIISTCANEKGGVPPLYSCPAPSA

>TaLTPVIIIa.1

AAPSSAQGTTTPADASGAVPSC---ASK------LVT-CAGYLNT-------TDTPPESC

CDPLKEAAT-------TQAACMCAILMNKAAL--QAFGVAPEQGVLLAKRCGV--TNDA-

----STCAK----------------

>OsLTPVIII.1

---------AVDTGAAAGVPSC---ASK------LVP-CGGYLNATAA-----P-PPASC

CGPLREAAA-------NETACLCAILTNKAAL--QAFGVAPEQGLLLAKRCGV--TTDA-

----SACAKSASSSATAAAAAAV--

>AtLTPVIII.1

------------------QTEC---VSK------IVP-CFRFLNT-------TTKPSTDC

CNSIKEAME-------KDFSCLCTIYNTPGLL--AQFNITTDQALGLNLRCGV--NTDL-

----SACSGTLILQDLRPLQL----

>AtLTPIX.1

-------------------HPCGRTFLS-----QLVP-CRPSVAPFST-----LPPNGLC

CAAIKTL----------GQPCLCVLAKGPPIV-----GVDRTLALHLPGKCSA--NFLP-

------CN-----------------

>AtLTPIX.2

QQEGLQQPPPPPMLPEEEVGGCSRTFFS-----QLIP-CRAAVAPFSP-----IPPTEIC

CSAVVTL----------GRPCLCLLANGPPLS-----GIDRSMALQLPQRCSA--NFPP-

------CDIIN--------------
